# Comparative proteomic analysis of human milk fat globules and paired membranes and mouse milk fat globules identifies core cellular systems contributing to mammary lipid trafficking and secretion

**DOI:** 10.3389/fmolb.2023.1259047

**Published:** 2023-12-18

**Authors:** Jayne F. Martin Carli, Monika Dzieciatkowska, Teri L. Hernandez, Jenifer Monks, James L. McManaman

**Affiliations:** ^1^ Section of Nutrition, Department of Pediatrics, University of Colorado Anschutz Medical Campus, Aurora, CO, United States; ^2^ Division of Reproductive Sciences, Department of Obstetrics and Gynecology, University of Colorado Anschutz Medical Campus, Aurora, CO, United States; ^3^ Department of Biochemistry and Molecular Genetics, University of Colorado Anschutz Medical Campus, Aurora, CO, United States; ^4^ College of Nursing, University of Colorado Anschutz Medical Campus, Aurora, CO, United States; ^5^ Division of Endocrinology, Metabolism, and Diabetes, Department of Medicine, University of Colorado Anschutz Medical Campus, Aurora, CO, United States

**Keywords:** molecular regulation of human milk secretion, milk fat globule (MFG), milk fat globule membrane (MFGM), mass spectrometry, comparative proteomics

## Abstract

**Introduction:** Human milk delivers critical nutritional and immunological support to human infants. Milk fat globules (MFGs) and their associated membranes (MFGMs) contain the majority of milk lipids and many bioactive components that contribute to neonatal development and health, yet their compositions have not been fully defined, and the mechanisms responsible for formation of these structures remain incompletely understood.

**Methods:** In this study, we used untargeted mass spectrometry to quantitatively profile the protein compositions of freshly obtained MFGs and their paired, physically separated MFGM fractions from 13 human milk samples. We also quantitatively profiled the MFG protein compositions of 9 pooled milk samples from 18 lactating mouse dams.

**Results:** We identified 2,453 proteins and 2,795 proteins in the majority of human MFG and MFGM samples, respectively, and 1,577 proteins in mouse MFGs. Using paired analyses of protein abundance in MFGMs compared to MFGs (MFGM-MFG; 1% FDR), we identified 699 proteins that were more highly abundant in MFGMs (MFGM-enriched), and 201 proteins that were less abundant in MFGMs (cytoplasmic). MFGM-enriched proteins comprised membrane systems (apical plasma membrane and multiple vesicular membranes) hypothesized to be responsible for lipid and protein secretion and components of membrane transport and signaling systems. Cytoplasmic proteins included ribosomal and proteasomal systems. Comparing abundance between human and mouse MFGs, we found a positive correlation (*R* ^2^ = 0.44, *p* < 0.0001) in the relative abundances of 1,279 proteins that were found in common across species.

**Discussion:** Comparative pathway enrichment analyses between human and mouse samples reveal similarities in membrane trafficking and signaling pathways involved in milk fat secretion and identify potentially novel immunological components of MFGs. Our results advance knowledge of the composition and relative quantities of proteins in human and mouse MFGs in greater detail, provide a quantitative profile of specifically enriched human MFGM proteins, and identify core cellular systems involved in milk lipid secretion.

## Introduction

Human milk is well appreciated to be the gold standard of nutrition for human infants (Victora et al., 2016). Currently, there is rapidly expanding interest in more fully elucidating how the interactions between individual milk components may make human milk greater than the sum of its parts (Christian et al., 2021; Raiten et al., 2023; Smilowitz et al., 2023). The broader “milk matrix” in which these individual milk components exist is thought to potentially contribute to both infant and maternal health. Many factors are expected to affect the milk matrix, including behavioral, environmental, structural, and organizational influences. Milk fat globules (MFG) are major structural, and organizationally complex, components of milk, and may be important contributors to the effects of the milk matrix. MFGs form a specialized lipid delivery system, providing nutritional components to support infant growth and development as well as immunological protection against disease. Their surrounding phospholipid trilayer membrane (milk fat globule membrane; MFGM) is unique to milk; infant formulas have traditionally provided lipids in the form of vegetable oil. Recent efforts have focused on adding MFGMs to infant formulas in recognition of the immunological and neurodevelopmental benefits associated with the protein, carbohydrate, and lipid constituents of MFGMs (Brink and Lonnerdal, 2020).

Data in laboratory and dairy animals have shown that MFG production begins with triacylglycerol synthesis in the endoplasmic reticulum (ER) (Stein and Stein, 1967; Kassan et al., 2013). Accumulating triacylglycerols are released into the cytoplasm where they are surrounded by an ER-derived phospholipid monolayer containing numerous attached or embedded proteins, resulting in formation of organelle structures referred to as cytoplasmic lipid droplets (CLD) (Dylewski et al., 1984; Walther and Farese, 2012). CLDs in mammary epithelial cells are coated with perilipin 2 (Plin2), which is thought to confer stability by protecting CLDs from lipolysis (Listenberger et al., 2007; Russell et al., 2011). CLDs can fuse to form larger CLDs in a process that is thought to be facilitated by cell death-inducing DNA fragmentation factor, alpha subunit-like effector A (Cidea), while concurrently trafficking toward the apical surface (Wooding, 1971; Stemberger and Patton, 1981; Wang et al., 2012; Wu et al., 2014; Barneda et al., 2015; Monks et al., 2016; Masedunskas et al., 2017) of the polarized luminal mammary epithelial cell (MEC). While moving intracellularly, CLDs can interact with other organelles, including the Golgi apparatus, mitochondria and casein-containing secretory vesicles (Wooding, 1971; Wooding, 1973; Stemberger et al., 1984; Wu et al., 2000; Mather et al., 2001; Honvo-Houéto et al., 2016). When they arrive at the apical cytoplasm, CLDs form contacts with the apical plasma membrane via interactions between CLD-coating Plin2, cytoplasmic xanthine dehydrogenase (Xdh; also known as xanthine oxidoreductase, Xor) and the transmembrane plasma membrane protein butyrophilin, subfamily 1, member A1 (Btn1a1) (Ishii et al., 1995; Keenan et al., 1995; Mather and Keenan, 1998; McManaman et al., 2002; Vorbach et al., 2002; Ogg et al., 2004a; Robenek et al., 2006; Jeong et al., 2009; Monks et al., 2016; Jeong et al., 2021). These proteins and their interactions allow for tight tethering, or docking, of CLDs to the apical plasma membrane, where they can continue to grow by fusion and protrude into the alveolar lumen (Dylewski et al., 1984; Monks et al., 2016; Masedunskas et al., 2017; Monks et al., 2022). Oxytocin release from the pituitary, which is driven by nipple stimulation and/or conditioned release in women (McNeilly et al., 1983), drives contraction of the surrounding myoepithelial cells which is proposed to cause membrane-tethered CLDs to be secreted into the alveolar lumen as MFGs by an apocrine mechanism (Kurosumi et al., 1968; Mather and Keenan, 1998; Mather et al., 2001; Masedunskas et al., 2017) that is incompletely understood. Cellular and biochemical studies indicate that the secretion process incorporates portions of the apical plasma membrane including proteins that form the CLD docking complex, parts of the cytosol, membrane elements of the endoplasmic reticulum, secretory and Golgi vesicles, and organellar transport machinery (Kurosumi et al., 1968; Wu et al., 2000; Honvo-Houéto et al., 2016; Wooding, 2023). Molecular details about how these cellular elements are integrated into MFGs and the precise role they play in the secretion process are limited. However, studies in mice indicate that formation of the CLD docking complex limits the amount of cytoplasm included in MFGs and enhances the efficiency of lipid secretion (Oftedal, 2012; Monks et al., 2016). Further functions of the CLD docking complex remain to be explored, and we anticipate that it may act as an intracellular scaffold and/or signaling hub to regulate overall milk production and secretion.

Milk fat secretion is critical to lactation success, as demonstrated in rodent models. Genetic disruption of the CLD synthetic machinery and docking complex components leads to poor offspring growth or starvation and death due to low milk consumption (Vorbach et al., 2002; Ogg et al., 2004b; Russell et al., 2011; Wang et al., 2012; Monks et al., 2016; Zhao et al., 2020; Jeong et al., 2021). Models targeting the machinery regulating triacylglycerol synthesis and CLD assembly impair glandular development and drive low milk fat production and secretion, decreasing milk caloric content (Smith et al., 2000; Beigneux et al., 2006; Russell et al., 2011; Wang et al., 2012; Suburu et al., 2014), and models targeting the MFG secretion machinery drive the production of extremely large and unstable MFGs, interfering with overall milk secretion (Vorbach et al., 2002; Ogg et al., 2004b; Monks et al., 2016; Jeong et al., 2021). Many of the mechanistic details of milk fat secretion have been worked out in dairy animals and/or model organisms by electron microscopy, immunohistochemistry and fluorescence microscopy in conjunction with genetic models and most recently, by elegant intravital imaging of glandular tissue (Wooding, 1971; Mather and Keenan, 1998; Monks et al., 2016; Masedunskas et al., 2017; Mather et al., 2019; Monks et al., 2020; Monks et al., 2022). Mammary tissue is difficult to obtain from humans for use with these methods, however, and our understanding of the regulation of human milk lipid production and secretion is therefore limited. As the milk lipid biosynthetic and secretory machinery are known to be retained on MFGs, we aimed to expand our understanding of human milk fat synthesis and secretion using a quantitative untargeted proteomic approach in MFGs. We also aimed to directly compare our findings from human samples to murine samples to identify how well the MFG production machinery is conserved between species, and therefore, how representative experimental murine models are to this process in humans. This is a particularly relevant question because the wide disparity between milk fat content in humans (3%–4%) (Ballard and Morrow, 2013) and mice (>20%) (Görs et al., 2009) could indicate divergent mechanisms of milk fat secretion.

Previous efforts to define the human MFGM proteome have identified the presence of MFG synthesis and docking complex protein homologs, including CIDEA, PLIN2, XDH/XOR and multiple BTN family members (Cavaletto et al., 2002; Fortunato et al., 2003; Liao et al., 2011; Spertino et al., 2012; Yang et al., 2015; Lu et al., 2016; Yang et al., 2016; Juvarajah et al., 2018; Zhang et al., 2021), in addition to over 400 other proteins. These studies have considered all proteins associated with MFGs to be membrane proteins. However, due to the apocrine mechanism of milk lipid secretion, MFGs also contain variable amounts of protein from cytoplasmic compartments (Patton and Huston, 1988). Distinguishing these from true membrane proteins can clarify which are required for CLD docking, envelopment and MFG secretion. We therefore directly compared the MFG proteome and the fractionated MFGM proteome from 13 women across early to mid-lactation. To isolate MFGMs, we utilized physical disruption (Mather, 2000; Reinhardt and Lippolis, 2008) rather than detergent-based disruption, as others have used for human MFGM analysis, to isolate membranes by centrifugation and limit the solubilization and loss of individual proteins from membrane complexes. Advancements in proteomics technology have allowed us to identify a far greater number of MFG and MFGM proteins than previously known, and our pathway analyses point toward potential regulators of milk fat synthesis and secretion.

## Materials and methods

### Human and mouse milk collection

Human milk (<1oz) was collected after an overnight fast in postpartum women, as part of a randomized controlled trial of diet composition in the control of gestational diabetes (Clinical Trials #NCT02244814). Milk collection protocols were approved by the Colorado Multiple Institutional Review Board (protocol #14–1358) as previously described (Hernandez et al., 2014; Hernandez et al., 2016; Martin Carli et al., 2020; Hernandez et al., 2023), and all participants gave their informed consent. Participants visits occurred at 2 weeks (5 samples), 2 months (3 samples) or 4–5 months postpartum (5 samples), following term deliveries (≥37 weeks). Milk was collected from a total of 9 participants. Two provided samples at both the 2 weeks and 4–5 months timepoints, and one participant provided milk at all three timepoints. Milk collections were not standardized with respect to the time of infant feeding or pumping. Samples were placed on ice in a cooler and transported to a study visit at the Clinical Translational Research Center at the University of Colorado Anschutz Medical Campus and then brought to the laboratory.

Mouse milk was collected from primiparous CD1 females from breeding colonies maintained in the AAALAC-Accredited (#00235) Center for Comparative Medicine at the University of Colorado Anschutz Medical Campus. The colony was housed under 14:10 h light:dark cycle at a temperature of 72 ± 2°F, humidity of 40% ± 10% and fed standard chow (Teklad/Envigo 2920X) and hyperchlorinated (2–5 ppm) water. Females were mated with CD1 males and then housed individually prior to parturition. The day a litter was first seen was counted as lactation day 1 (L1). Dams were allowed to nurse their natural born litter (litters were not standardized, avg: 12 ± 2 pups/litter). Milk samples were collected from 18 dams at L9-11, after 3 h of separation from pups, as previously described (Monks et al., 2022). Briefly, intraperitoneal (IP) xylazine was given at a dose of 8 mg/kg. When the mouse was relaxed enough to have ceased ambulation around the cage (about 5 min), the milking procedure was initiated. The mouse was picked up, and with gentle hand-restraint, a single dose of oxytocin (0.25 IU, 0.12 mL in sterile saline) was administered IP. Milk let-down occurred within 1 min and milk removal was started. Our standard milking apparatus, attached to house vacuum, was used. Hand restraint was used throughout the milking procedure. Milk was collected and processed at room temperature to avoid changes in protein segregation between phases. All animal experiments and procedures were approved by the University of Colorado Anschutz Medical Campus’ Institutional Animal Care and Use Committee on protocol 00985 (PI: McManaman).

### MFG and MFGM isolation

**FIGURE 1:**
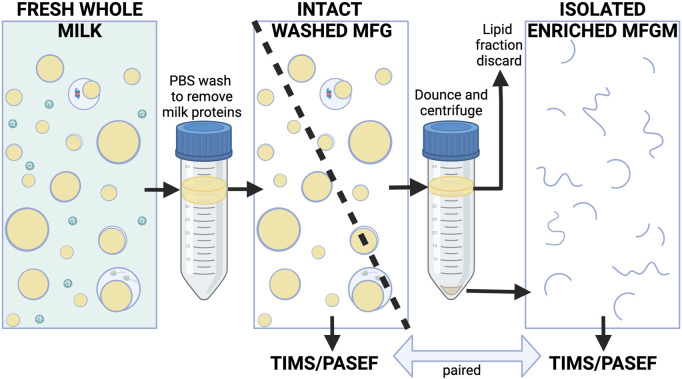
Simplified conceptual diagram of milk fractionation for proteomic analysis of human MFG fractions. Freshly collected and floated MFGs were gently washed of milk proteins with PBS. Intact, floated washed MFG from each subject were divided into two aliquots. One aliquot of each sample was used for proteomic analysis of whole MFGs, the other aliquot of each sample was Dounce-homogenized and centrifuged to pellet the enriched MFGM fraction. The MFGM fraction was sent for proteomic analysis along with its paired washed MFG fraction (see Methods for details). Whey proteins and casein micelles are shown in green. Lipids are yellow. Membranes are blue. MFGM fractions from some species are known to contain variable quantities of cytoplasmic components (Wooding and Kemp, 1975), however they are presented in a simplified manner here. Created with BioRender.com.

Intact MFGs from fresh human and murine milk samples were isolated according to procedures previously described (Monks et al., 2022) which were informed by established methods (Patton and Huston, 1986; Wu et al., 2000; Monks et al., 2016). Milk was protected from freezing to avoid damaging membranes. Briefly, whole milk samples were gently combined with ∼10 volumes of PBS and centrifuged at 1,500 xg at room temperature for 10 min to float MFGs as described by Patton and Huston (Patton and Huston, 1986). To isolate MFGs from small volumes of highly viscous mouse milk, samples were mixed 1:1 with 10% sucrose and layered under PBS for this first centrifugation wash, which minimized adhesion to tubes and pipet tips, and subsequently allowed the lower density MFGs to float to the top. Two human samples were treated this way, however this appeared to contribute to sample loss, so the remaining human samples were not mixed 1:1 with 10% sucrose. Floated MFGs from individual women were collected, gently washed and refloated twice by mixing with 14 mLs of PBS and centrifuging at 1,500 xg, at room temperature for 10 min. Washed intact human MFGs were divided into two aliquots and then frozen at −80°C. One aliquot was processed directly for mass spectrometry analysis without further fractionation. The second aliquot was used to prepare MFGMs using procedures previously established for isolating bovine MFGMs (Reinhardt and Lippolis, 2008). Briefly, frozen MFGs were thawed on ice, Dounce homogenized (100 strokes) and centrifuged at 22,000 xg for 20 min at 4°C. Pelleted membranes (MFGMs) were stored at −80°C prior to proteomic analysis. We did not obtain enough starting material to efficiently isolate MFGMs from murine milk samples. This process is illustrated in Figure 1.

### Liquid chromatography—tandem mass spectrometry (LC-MS/MS)

Washed MFG or isolated MFGM samples were precipitated with 10% trichloroacetic acid for 2 h at −20°C. The precipitated protein samples were pelleted by centrifugation at 14,000 xg for 20 min at 4°C, rinsed in ice-cold acetone and centrifuged again. The pellet was air-dried and solubilized in 5% SDS, 100 mM DTT in 100 TEAB. The samples were digested using the S-Trap filter (Protifi, Huntington, NY) according to the manufacturer’s procedure. Briefly, samples were reduced with 10 mM DTT at 55°C for 30 min, cooled to room temperature, and then alkylated with 25 mM iodoacetamide in the dark for 30 min. Next, a final concentration of 1.2% phosphoric acid and then six volumes of binding buffer (90% methanol; 100 mM triethylammonium bicarbonate, TEAB; pH 7.1) were added to each sample. After gently mixing, the protein solution was loaded to a S-Trap filter, spun at 1,000 xg for 1 min, and the flow-through collected and reloaded onto the filter. This step was repeated three times, and then the filter was washed with 200 μL of binding buffer 3 times. Finally, 1 μg of sequencing-grade trypsin (Promega) in 150 μL of digestion buffer (50 mM TEAB) were added onto the filter and digestion was carried out at 37°C for 6 h. To elute peptides, three stepwise buffers were applied, with 100 μL of each with one more repeat, including 50 mM TEAB, 0.2% formic acid in H_2_O, and 50% acetonitrile and 0.2% formic acid in H_2_O. The peptide solutions were pooled, lyophilized and resuspended in 100 μL of 0.1% FA.

20 μL of each sample was loaded onto individual Evotips for desalting and then washed with 20 μL 0.1% FA followed by the addition of 100 μL storage solvent (0.1% FA) to keep the Evotips wet until analysis. The Evosep One system (Evosep, Odense, Denmark) was used to separate peptides on a Pepsep column, (150 um inter diameter, 15 cm) packed with ReproSil C18 1.9 um, 120A resin. The system was coupled to the timsTOF Pro mass spectrometer (Bruker Daltonics, Bremen, Germany) via the nano-electrospray ion source (Captive Spray, Bruker Daltonics). The mass spectrometer was operated in PASEF mode (TIMS/PASEF). The ramp time was set to 100 ms and 10 PASEF MS/MS scans per topN acquisition cycle were acquired. MS and MS/MS spectra were recorded from m/z 100 to 1700. The ion mobility was scanned from 0.7 to 1.50 Vs/cm^2^. Precursors for data-dependent acquisition were isolated within ±1 Th and fragmented with an ion mobility-dependent collision energy, which was linearly increased from 20 to 59 eV in positive mode. Low-abundance precursor ions with an intensity above a threshold of 500 counts but below a target value of 20,000 counts were repeatedly scheduled and otherwise dynamically excluded for 0.4 min.

### Database searching and protein identification

MS/MS spectra were extracted from raw data files and converted into .mgf files using MS Convert (ProteoWizard, Ver. 3.0). Peptide spectral matching was performed with Mascot (Ver. 2.6) against the Uniprot human and mouse databases. Mass tolerances were ±15 ppm for parent ions, and ±35 ppm for fragment ions. Trypsin specificity was used, allowing for one missed cleavage. Met oxidation, protein N-terminal acetylation and peptide N-terminal pyroglutamic acid formation were set as variable modifications with Cys carbamidomethylation set as a fixed modification.

Scaffold (version 5.0, Proteome Software, Portland, OR, United States) was used to validate MS/MS based peptide and protein identifications. Peptide identifications were accepted if they could be established at greater than 95.0% probability as specified by the Peptide Prophet algorithm. Protein identifications were accepted if they could be established at greater than 99.0% probability and contained at least two identified unique peptides. Proteins are identified in the text by their official gene names and symbols, as these were utilized for pathway analyses described below. In instances where there are multiple protein products encoded by a single gene, the major product is specified.

### Statistical analyses

Milk collections were treated as independent samples, even though a subset of participants provided repeated samples. This is because milk fat composition is largely affected by diet and collection variables that we could not account for, such as whether foremilk or hindmilk was collected, or time since last breast emptying. Statistical analyses were conducted as described using Graphpad Prism 9.5.1 and Metaboanalyst 5.0 (https://www.metaboanalyst.ca/home.xhtml). GO cellular component analysis was conducted using DAVID (https://david.ncifcrf.gov/home.jsp) from NIAID/NIH (Huang et al., 2009; Sherman et al., 2022).

### Pathway analyses

Gene symbols of the proteins detected in ≥50% or 100% of MFG or MFGM samples, for human, or ≥50% or 100% of murine MFG samples were utilized for pathway enrichment analysis using Metascape (https://metascape.org/gp/index.html#/main/step1). This tool queries multiple different ontology sources, and minimizes redundancy by clustering related pathway terms (Zhou et al., 2019). First, all statistically enriched terms were identified (can be GO/KEGG terms, canonical pathways, hall mark gene sets, etc., based on the default choices under Express Analysis), accumulative hypergeometric *p*-values and enrichment factors were calculated and used for filtering. Remaining significant terms were then hierarchically clustered into a tree based on Kappa-statistical similarities among their gene memberships. Then 0.3 kappa score was applied as the threshold to cast the tree into term clusters. To visualize our results in the context of cellular mechanisms, we utilized Reactome’s (Fabregat et al., 2018) pathway diagram viewer (https://reactome.org/, version 3.7) with our gene symbol lists.

### Data availability

Proteomics datasets were uploaded into the MassIVE Center for Computational Mass Spectrometry entitled “Proteomic Analysis of Paired Human Milk Fat Globules and Milk Fat Globule Membranes.” (MassIVE MSV000092892) and “Proteomic Analysis of Mouse Milk Fat Globules” (MassIVE MSV000092915).

## Results

**FIGURE 2:**
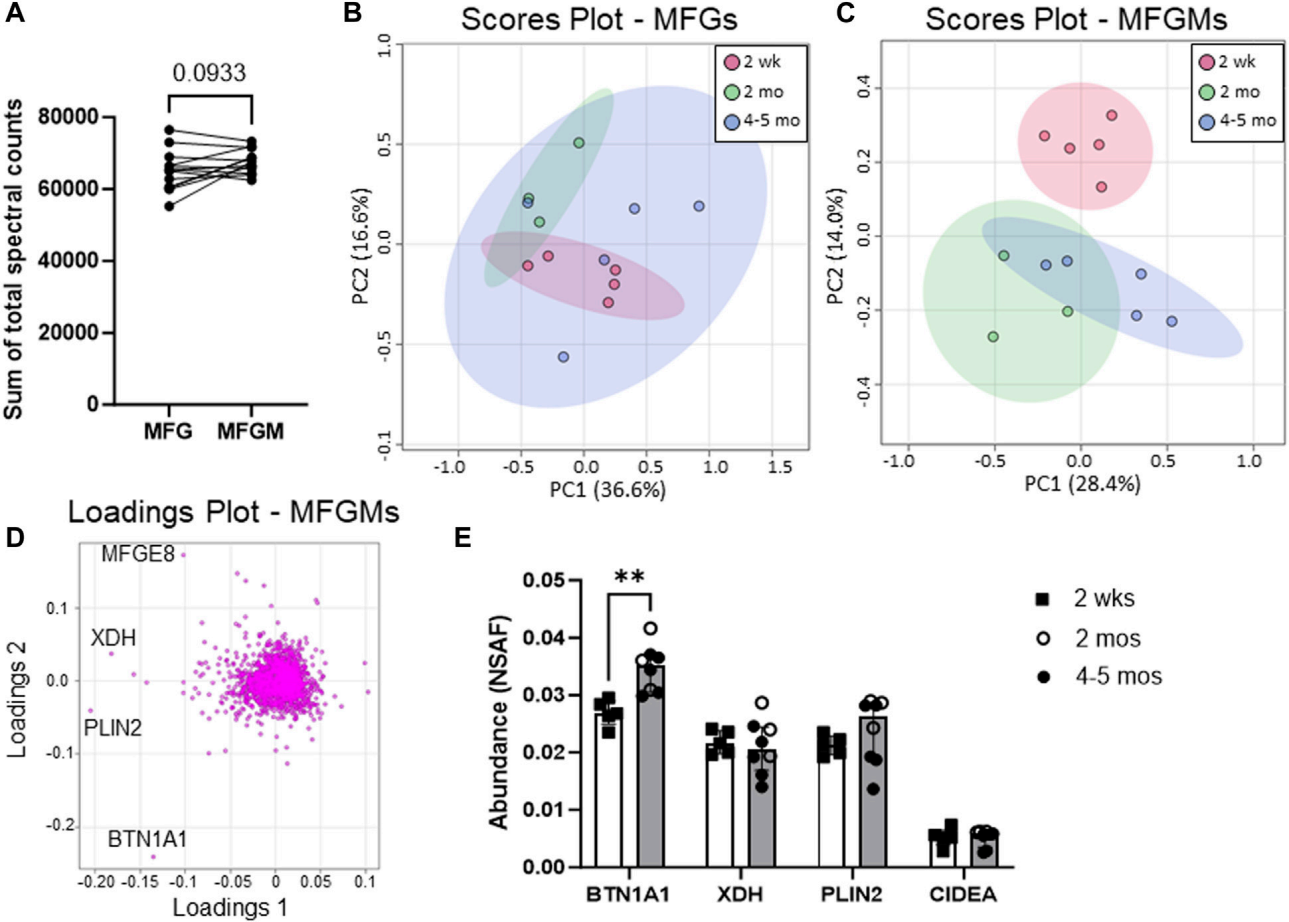
Human MFG and MFGM protein abundance over time. Sum of human MFG and MFGM spectral counts by LC-MS/MS **(A)**. Principal components analysis of 2,453 MFG-associated **(B)** and 2,795 MFGM-associated **(C)** proteins separated by time postpartum: red = 2 weeks, green = 2 months, blue = 4–5 months. Pareto scaling was used to limit the effects of large fold changes found in low abundance proteins. Loadings of MFGM-associated protein PCA **(D)**. Abundance of known CLD docking proteins BTN1A1, XDH, PLIN2 and CIDEA **(E)**. Open bars = 2 weeks (*n* = 5), gray bars = 2–5 months, open circles = 2 months (*n* = 3), closed circles = 4–5 months (*n* = 5). Multiple nonparametric (Mann-Whitney), unpaired t-tests, adjusted for multiple testing with the Holm-Šídák method. **p < 0.01.

In this study, we defined quantitative proteomic profiles of MFG and MFGM pairs from 13 women across early (2 weeks postpartum, *n* = 5) to mature lactation (2 months postpartum, *n* = 3, and 4–5 months postpartum, *n* = 5) by LC-MS/MS (Figure 1). The sum of the total spectral counts was not different between MFG and MFGM proteins, indicating effective sample preparation and similar loading between sample types (Figure 2A). We included in our analyses the 2,933 proteins which were detected in ≥50% of replicates in one or both groups to identify low abundance proteins which might provide important biological data in aggregate. Using a threshold of proteins detected in 100% of replicates in one or both sample types, we identified 1,812 proteins. We first considered the possibility that MFG and/or MFGM proteins from late-transitional/early mature milk at 2 weeks postpartum could differ from those found in mature milk from 2 to 5 months postpartum, especially as bovine MFGM proteins have been shown to change from colostrum to mature milk (Reinhardt and Lippolis, 2008). Using unbiased principal components analysis (PCA), we found that MFG proteins (Figure 2B) could not be distinguished across timepoints by the first five principal components (79.7% of total variance). However, MFGM proteins at transitional vs. mature timepoints were separated across PC2 (14.0%; Figure 2C). BTN1A1 was the biggest driver of this separation, with a loading score of −0.23 (Figure 2D). The abundance of BTN1A1 increased from 0.027 (0.026–0.028) normalized spectral abundance factor [med (IQR); NSAF] in early lactation to 0.034 (0.031–0.037) NSAF, in mature lactation (adj. *p* < 0.01; Figure 2E). Other components of the CLD docking complex, XDH/XOR, PLIN2 and CIDEA, which are increased in the transition from bovine colostrum to mature milk (Reinhardt and Lippolis, 2008), were not increased during the transition from early to mature human lactation (Figure 2E). When the entire proteomic dataset was considered, we did not find statistically significant differential abundances between early and mature lactation (Supplementary Figure S1). Many factors, including time since last feeding and time respective to the feeding/pumping bout are expected to affect CLD docking and envelopment (Masedunskas et al., 2017; Mather et al., 2019) and therefore, protein composition of the MFGM. We are unable to account for these collection details in the current study, which did not standardize these factors, and we do not have access to samples from the colostrum phase. As we were underpowered to investigate temporal changes across lactation, we combined all timepoints for subsequent analyses to maximize our statistical power.

**FIGURE 3:**
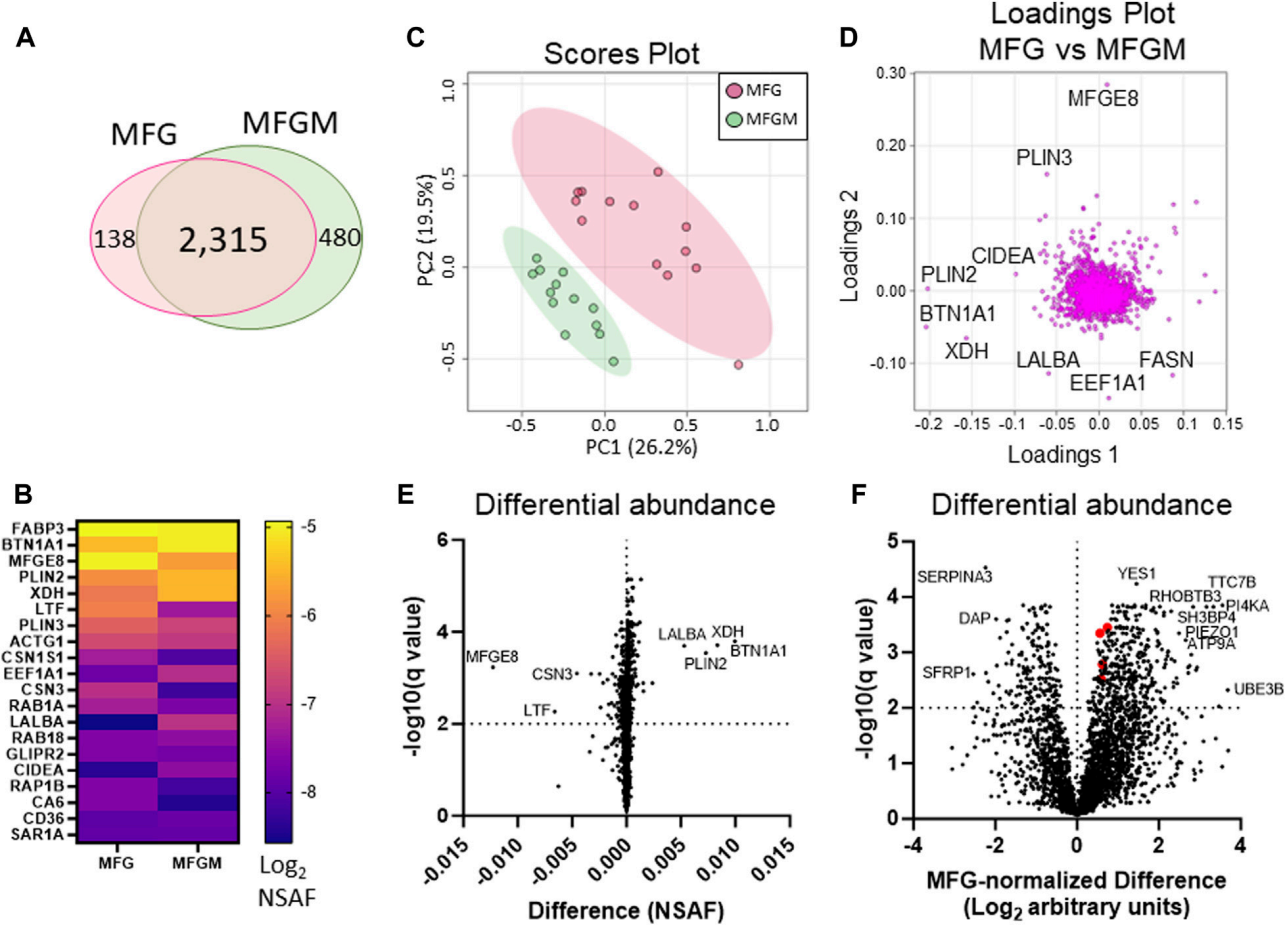
Differential protein abundance between human MFGs and MFGMs. Venn diagram of shared and distinct human MFG- and MFGM-associated proteins **(A)**. Abundance of the top 20 human MFG- and MFGM-associated proteins [**(B)**, Log_2_ NSAF], sorted by means of MFG and MFGM values combined. Principal components analysis of MFG-associated (red) and MFGM-associated (green) proteins **(C)**. Pareto scaling was used to limit the effects of large fold changes found in low abundance proteins. Loadings of MFG- and MFGM-associated protein PCA **(D)**. Multiple paired t-tests of MFG-vs MFGM-associated proteins, calculated by absolute difference [MFGM-MFG, **(E)**] or by differences in Log_2_-transformed, MFG-normalized values, [Log_2_(MFGM/AvgMFG)-Log_2_(MFG/AvgMFG); **(F)]** with FDR correction for multiple comparisons (1%). BTN1A1, XDH, PLIN2 and CIDEA are labeled red in **(F)**.

We identified 2,315 proteins that were common between MFGs and their membrane fractions, as well as 138 unique proteins in the MFG samples and 480 unique proteins in the MFGM samples. (Figure 3A). A heatmap of the 20 most abundant proteins across both sample types is shown in Figure 3B and values are listed in Table 1. Values for all proteins detected are listed in Supplementary Table S1. Although differences in sequence coverage, due to variation in tryptic sites and ionization efficiency, can limit stoichiometric analysis of proteomic data, we obtained high coverage (>60%) of most major proteins in the MFG and MFGM fractions (Supplementary Figure S2A). The abundances of BTN1A1, PLIN2 and XDH/XOR were greater than other MFGM proteins with the exception of fatty acid binding protein 3 (FABP3), which had a relative abundance similar to that of BTN1A1. In the MFG fraction, FABP3 and milk fat globule EGF and factor V/VIII domain containing (MFGE8, major protein product: lactadherin) were the most abundant proteins, followed by BTN1A1, PLIN2, lactotransferrin (LTF), XDH/XOR and perilipin 3 (PLIN3). We found that of previously described CLD docking proteins, both in MFG samples and isolated MFGM proteins, BTN1A1 was the most abundant protein, followed by PLIN2 and XDH/XOR. These three proteins were more highly abundant than CIDEA, which is also implicated in CLD docking (Monks et al., 2016). In aggregate, these four CLD docking complex proteins represented 6.8% of spectra detected across all 2,933 proteins, which is consistent with their proposed structural role in mediating CLD-apical plasma membrane contacts that facilitate milk lipid secretion (Monks et al., 2022).

**TABLE 1:** Top 20 most abundant MFG and MFGM-associated proteins.

Identified protein	Gene symbol	MFG NSAF Avg (SD)	MFGM NSAF Avg (SD)	All NSAF Avg (SD)
Fatty acid-binding protein, heart	FABP3	0.038 (0.0176)	0.0317 (0.0102)	0.0348 (0.0145)
Butyrophilin subfamily 1 member A1	BTN1A1	0.0217 (0.0042)	0.0317 (0.0051)	0.0267 (0.0069)
Lactadherin	MFGE8	0.0308 (0.0096)	0.0185 (0.004)	0.0247 (0.0095)
Perilipin-2	PLIN2	0.0155 (0.005)	0.0228 (0.0047)	0.0192 (0.006)
Xanthine dehydrogenase/oxidase	XDH	0.0129 (0.0031)	0.0213 (0.0039)	0.0171 (0.0055)
Lactotransferrin	LTF	0.0137 (0.0082)	0.007 (0.003)	0.0103 (0.0069)
Perilipin-3	PLIN3	0.0109 (0.0036)	0.009 (0.0032)	0.0099 (0.0035)
Actin, cytoplasmic 2	ACTG1	0.0096 (0.0012)	0.0082 (0.0019)	0.0089 (0.0017)
Alpha-S1-casein	CSN1S1	0.0075 (0.0046)	0.0053 (0.0033)	0.0064 (0.0041)
Elongation factor 1-alpha 1	EEF1A1	0.005 (0.0021)	0.0074 (0.0016)	0.0062 (0.0022)
Kappa-casein	CSN3	0.0083 (0.004)	0.0038 (0.002)	0.006 (0.0039)
Ras-related protein Rab-1A	RAB1A	0.0064 (0.0017)	0.0054 (0.0013)	0.0059 (0.0016)
Alpha-lactalbumin	LALBA	0.0032 (0.0016)	0.0085 (0.0035)	0.0059 (0.0038)
Ras-related protein Rab-18	RAB18	0.0051 (0.0014)	0.0054 (0.0012)	0.0052 (0.0013)
Golgi-associated plant pathogenesis-related protein 1	GLIPR2	0.0049 (0.0021)	0.0043 (0.0011)	0.0046 (0.0016)
Cell death activator CIDE-A	CIDEA	0.0036 (0.0016)	0.0052 (0.0015)	0.0044 (0.0017)
Ras-related protein Rap-1b	RAP1B	0.0052 (0.0018)	0.0036 (0.0009)	0.0044 (0.0017)
Carbonic anhydrase 6	CA6	0.0055 (0.0034)	0.0031 (0.0018)	0.0043 (0.0029)
Platelet glycoprotein 4	CD36	0.0041 (0.0016)	0.0045 (0.0011)	0.0043 (0.0014)
GTP-binding protein SAR1a	SAR1A	0.0041 (0.0018)	0.0043 (0.0011)	0.0042 (0.0015)

Abundance of the top 20 human MFG- and MFGM-associated proteins, sorted by means of MFG and MFGM values combined, displayed as a heatmap in Figure 2B.

We next compared MFG and MFGM proteins by unbiased PCA and identified distinctly different proteomes in these fractions (Figure 3C). As expected, the loadings contributing to this distinction primarily consisted of proteins implicated in CLD docking; XDH/XOR, BTN1A1, PLIN2 and CIDEA (Figure 3D). To identify possible additional components of the human CLD docking complex, we calculated differential protein abundances between sample types by volcano plots, comparing differences in both absolute NSAF values (Figure 3E) to assess highly abundant proteins, and values normalized to average MFG values (MFG-normalized; Figure 3F), to assess proteins with low abundance. We considered proteins that were more highly abundant in the MFGM fraction to be MFGM-enriched and proteins more highly abundant in the whole MFG proteome to be cytoplasmic. The top 10 proteins with the largest absolute and MFG-normalized differences between sample types, both MFGM-enriched and cytoplasmic, are listed in Table 2. All differentially abundant proteins by absolute and MFG-normalized differences are listed in Supplementary Tables S2, S3, respectively. We identified 900 proteins which were statistically significant by absolute difference (699 MFGM-enriched and 201 cytoplasmic) and 537 by MFG-normalized difference (371 MFGM-enriched and 166 cytoplasmic).

**TABLE 2:** Most differentially abundant proteins in human MFG and MFGM samples.

A. Absolute differences					
Enrichment	Gene Symbol	MFGM Mean (SD) (NSAF)	MFG Mean (SD) (NSAF)	Difference (SE) (NSAF)	q value
MFGM-enriched	BTN1A1	0.0317 (0.0051)	0.0217 (0.0042)	0.01 (0.0013)	0.00016
	XDH	0.0213 (0.0039)	0.0129 (0.0031)	0.0083 (0.0011)	0.00020
	PLIN2	0.0228 (0.0047)	0.0155 (0.005)	0.0073 (0.0011)	0.00029
	LALBA	0.0085 (0.0035)	0.0032 (0.0016)	0.0053 (0.0007)	0.00020
	EEF1A1	0.0074 (0.0016)	0.005 (0.0021)	0.0024 (0.0004)	0.00130
	CIDEA	0.0052 (0.0015)	0.0036 (0.0016)	0.0016 (0.0003)	0.00193
	ABCG2	0.0027 (0.0012)	0.0012 (0.0005)	0.0015 (0.0003)	0.00130
	CYB5A	0.0025 (0.0005)	0.0012 (0.0005)	0.0013 (0.0001)	0.00001
	S100A1	0.0023 (0.0005)	0.001 (0.0004)	0.0012 (0.0001)	0.00006
	TLR2	0.0021 (0.0005)	0.001 (0.0003)	0.0011 (0.0001)	0.00017
Cytoplasmic	MFGE8	0.0185 (0.004)	0.0308 (0.0096)	−0.0123 (0.002)	0.00058
	LTF	0.007 (0.003)	0.0137 (0.0082)	−0.0066 (0.0017)	0.00541
	CSN3	0.0038 (0.002)	0.0083 (0.004)	−0.0046 (0.0008)	0.00080
	ENO1	0.0021 (0.0013)	0.0054 (0.0029)	−0.0033 (0.0006)	0.00082
	RALB	0.002 (0.0008)	0.0044 (0.002)	−0.0025 (0.0004)	0.00082
	CA6	0.0031 (0.0018)	0.0055 (0.0034)	−0.0024 (0.0006)	0.00439
	GAPDH	0.0024 (0.0012)	0.0046 (0.002)	−0.0022 (0.0004)	0.00129
	CDC42	0.0025 (0.0006)	0.0043 (0.0011)	−0.0018 (0.0003)	0.00109
	CEL	0.0025 (0.0006)	0.0043 (0.0011)	−0.0017 (0.0004)	0.00649
	RAP1B	0.0036 (0.0009)	0.0052 (0.0018)	−0.0016 (0.0004)	0.00373

B. MFG-normalized differences					
	Gene Symbol	MFG-normalized MFGMMean (SD) (AU)	MFG-normalized MFGMean (SD) (AU)	Difference of Log2 MFG normalized values Mean (SE) (AU)	q value
MFGM-enriched	UBE3B	33.73 (16.9)	1 (1.56)	3.68 (0.27)	0.00476
	TTC7B	13.76 (6.17)	1 (0.69)	3.55 (0.3)	0.00014
	NBAS	10.87 (7.53)	1 (1.82)	3.47 (0.59)	0.00936
	PI4KA	9.35 (4.17)	1 (0.88)	3.33 (0.35)	0.00015
	SH3BP4	6.79 (2.33)	1 (0.85)	3.16 (0.34)	0.00015
	TRPM4	10.49 (6.56)	1 (1.61)	2.93 (0.3)	0.00185
	ITPR1	4.57 (2.01)	1 (1.35)	2.91 (0.52)	0.00205
	RHOBTB3	7.13 (2.23)	1 (0.8)	2.83 (0.27)	0.00015
	WFS1	7.45 (4.05)	1 (1.15)	2.79 (0.41)	0.00109
	SPTBN1	4.58 (1.79)	1 (1.08)	2.75 (0.51)	0.00173
Cytoplasmic	SFRP1	0.19 (0.33)	1 (1.18)	−2.54 (0.34)	0.00246
	MRAS	0.16 (0.27)	1 (0.65)	−2.53 (0.44)	0.00977
	C9	0.16 (0.2)	1 (1.02)	−2.45 (0.33)	0.00797
	SERPINA3	0.19 (0.17)	1 (0.91)	−2.24 (0.15)	0.00003
	CA2	0.22 (0.28)	1 (0.62)	−2.23 (0.36)	0.00233
	GSS	0.21 (0.24)	1 (0.89)	−2.17 (0.39)	0.00337
	GCHFR	0.3 (0.47)	1 (0.75)	−2.05 (0.38)	0.00542
	DAP	0.16 (0.23)	1 (0.94)	−1.98 (0.1)	0.00025
	IGHG2	0.15 (0.1)	1 (1.86)	−1.97 (0.34)	0.00172
	PPT1	0.23 (0.32)	1 (1.26)	−1.92 (0.25)	0.00395

Top 10 most differentially abundant proteins in the MFGM (upper values) and cytoplasmic fraction of the MFG (lower values) calculated by absolute difference (A) or MFG-normalized differences (B).

**FIGURE 4:**
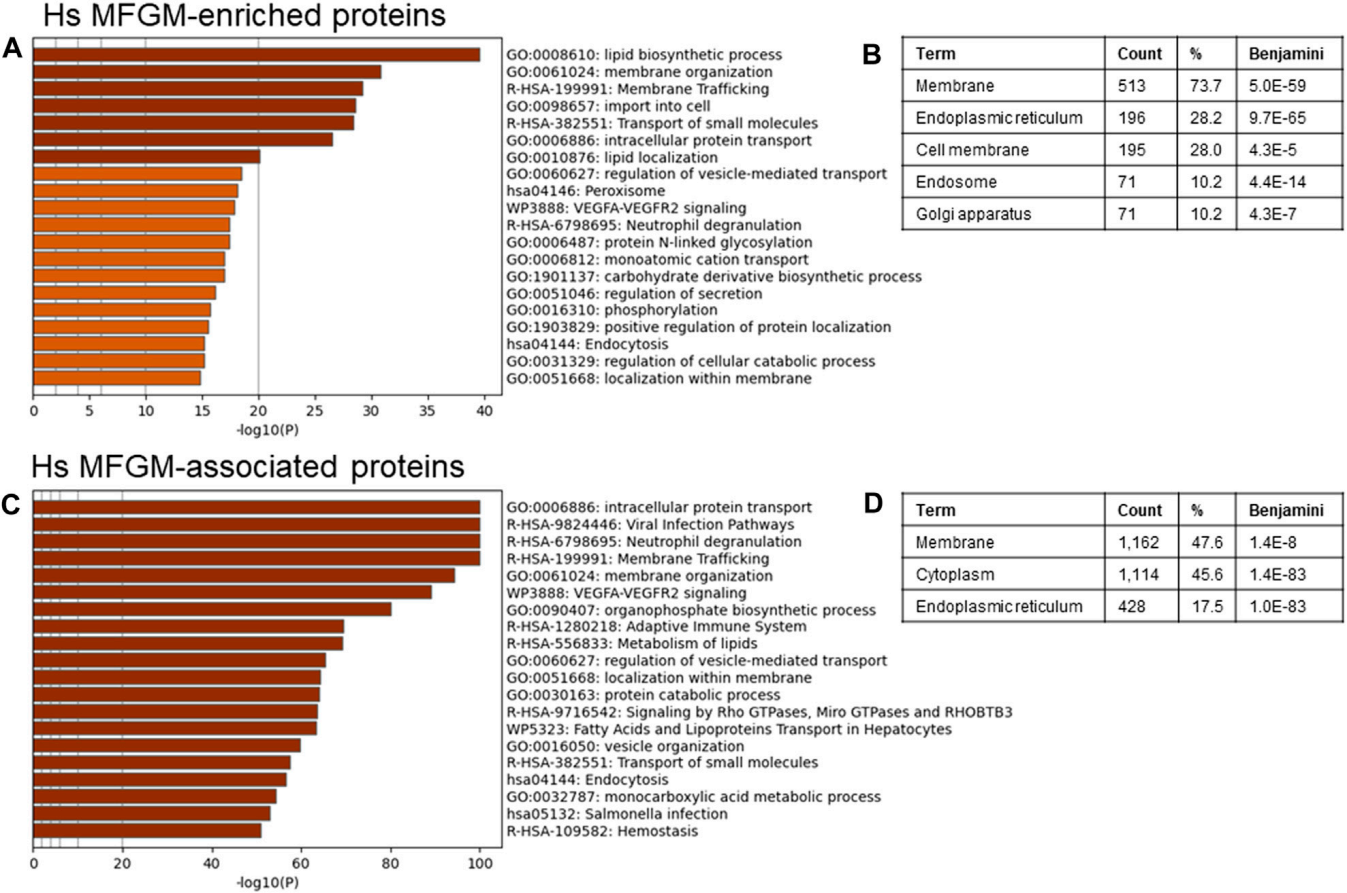
Pathway analyses of human MFGM-enriched and MFGM-associated proteins. Metascape pathway enrichment analysis **(A)** and GO Cellular components analysis **(B)** of MFGM-enriched proteins, calculated by difference. Top 20 pathway clusters are shown in **(A)**. Metascape pathway enrichment analysis **(C)** and GO Cellular components analysis **(D)** of all MFGM-associated proteins. Top 20 pathway clusters are shown in **(C)**.

To identify possible functional properties of MFGM-enriched proteins, we conducted unbiased pathway analysis using Metascape (Zhou et al., 2019), which comprehensively utilizes KEGG Pathway, GO Biological Processes, Reactome Gene Sets, Canonical Pathways, CORUM, WikiPathways, and PANTHER Pathway as ontology sources. We report pathways identified using the threshold of proteins present in 50% of samples, and in Supplementary Figures S3–S8 show the top 100 pathways identified using a threshold of proteins present in 100% of samples, to eliminate pathways identified due to low abundance and/or low confidence proteins. Differentially abundant proteins identified in Figure 3E were utilized to avoid losing datapoints undetected in MFGs, and therefore with a denominator of 0 when conducting normalization to MFG values. Using this approach, we found that human MFGM-enriched proteins (Figure 4A—top 20 pathway clusters, Supplementary Figure S3—top 100 pathway clusters and Supplementary Table S4—all pathways) identified highly enriched (−log_10_ *p* < 14) pathway clusters related to lipid metabolism and localization (lipid biosynthetic process and lipid localization), endoplasmic reticulum (protein N-linked glycosylation) and intracellular trafficking, as expected. Intracellular trafficking-related pathway clusters include membrane organization, membrane trafficking, import into cell, transport of small molecules, intracellular protein transport, regulation of vesicle-mediated transport, regulation of secretion, positive regulation of protein localization and localization within membrane. Interestingly, this analysis also identified neutrophil degranulation and VEGFA-VEGFR2 signaling pathway clusters. Using GO cellular component analyses, we find that MFGM-enriched proteins correspond to significant contributions from the cell membrane and organelle sub-compartments, including the ER, Golgi apparatus and endosome (Figure 4B).

Not all proteins in the MFGM fraction were significantly elevated in comparison to the whole MFG, due to similar distribution across membrane and cytoplasmic compartments. We considered the possibility that the totality of proteins (*n* = 2,795) associated with the MFGM may reflect the cellular process involved in MFG formation and secretion. When all proteins in the MFGM fraction (MFGM-associated proteins) were included in pathway analysis, we identified highly enriched clusters (−log_10_ *p* < 50) related to pathways and processes corresponding to metabolism of lipids, intracellular trafficking and organization (intracellular protein transport, membrane trafficking, membrane organization, regulation of vesicle-mediated transport, localization within membrane, fatty acids and lipoproteins transport in hepatocytes, vesicle organization and endocytosis) and the immune system (viral infection pathways, neutrophil degranulation, adaptive immune system and *salmonella* infection). VEGF-VEGFR2 and Rho and Miro GTPases and RHOBTB3 signaling, protein catabolic process, and monocarboxylic acid metabolic process were also present (Figure 4C–top 20 pathway clusters, Supplementary Figure S4—top 100 pathway clusters and Supplementary Table S5—all pathways). GO cellular component analyses of MFGM-associated proteins show significant contributions of membrane and cytoplasmic proteins as well as those from the ER (Figure 4D). Collectively, these observations are consistent with the initial proteomic studies of mouse MFGM which suggest that MFGM proteins are derived in part from the ER (Wu et al., 2000), and further indicate that proteins within specific ER sub-compartments, secretory granules and vesicular membranes are major contributors to the human MFGM proteome.

**FIGURE 5:**
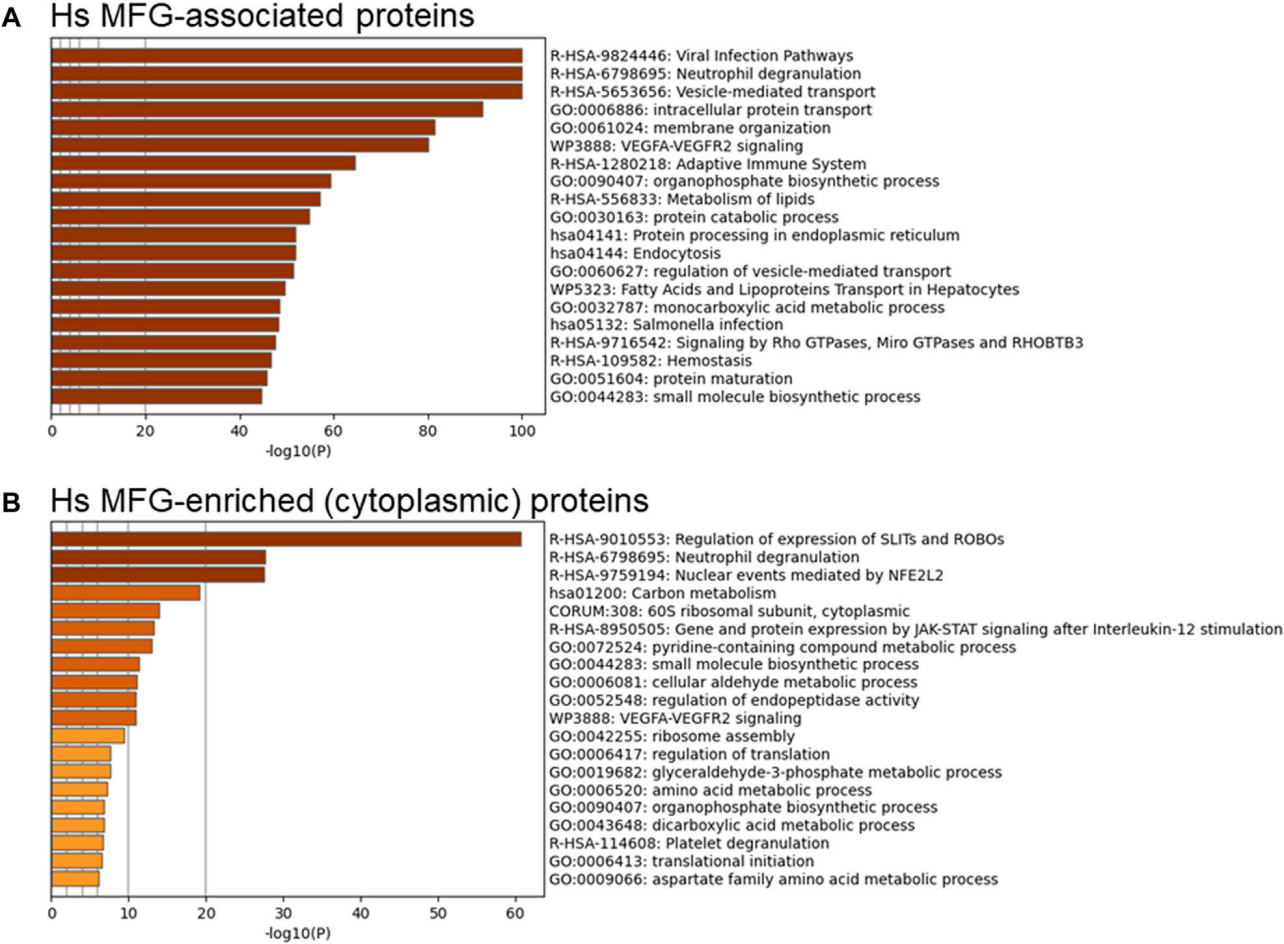
Pathway analyses of human MFG-associated proteins. Metascape pathway enrichment analysis of MFG-associated proteins **(A)** and MFG-enriched (cytoplasmic) proteins **(B)**. Top 20 pathway clusters are shown.

Although the MFG and MFGM proteomes were distinct by principal components analysis, we aimed to determine which pathways were found in common between MFG-associated and MFGM-associated proteins and which were unique. Using all MFG-associated proteins (*n* = 2,453; Figure 5A—top 20 pathway clusters, Supplementary Figure S5—top 100 pathway clusters and Supplementary Table S6—all pathways) for pathway analysis, we identified pathway clusters which we identified using the MFGM proteome, including neutrophil degranulation, vesicle-mediated transport, VEGFA-VEGFR2 signaling, and Rho GTPases, Miro GTPases and RHOBTB3, in addition to lipid metabolism, endoplasmic reticulum and intracellular trafficking-related pathway clusters. Therefore, pathway analysis of MFG-associated pathways identifies pathways active both in the endoplasmic reticulum and apical plasma membranes as well as the encapsulated cytoplasm.

To obtain a clearer understanding of proteins found exclusively in the encapsulated cytoplasm, we also conducted pathway analysis of the 201 proteins which were differentially more abundant in the MFG compared to the MFGM (Figure 5B–top 20 pathway clusters, Supplementary Figure S6—top 100 pathway clusters and Supplementary Table S7—all pathways). Translational pathways were heavily represented in the resulting pathway clusters identified (60S ribosomal subunit, cytoplasmic, ribosome assembly, regulation of translation and translational initiation). Other pathway clusters indicate overrepresentation of ribosomal proteins and also suggest the presence of proteasomal proteins. SLITs and ROBOs have been implicated in development of both the normal mammary gland and breast cancer (Marlow et al., 2010; Macias et al., 2011; Harburg et al., 2014; Ballard Mimmi et al., 2015; Zhao et al., 2016) and ROBO1 signaling has been shown to mediate differentiation for milk secretion (Cazares et al., 2021). However, the proteins in this pathway which are present in our MFG-enriched list include 16 proteins which are components of the proteasome, 30 proteins which are ribosomal subunits and none which suggest that MFG secretion regulates specific expression of the SLIT/ROBO pathway. Similarly, the identification of the pathway cluster, “nuclear events mediated by NFE2L2” appears to be driven by the same 16 proteasomal proteins. It is not clear if loss of these cellular components in cytoplasmic crescents during MFG secretion affects the function of the milk secreting cell.

**FIGURE 6:**
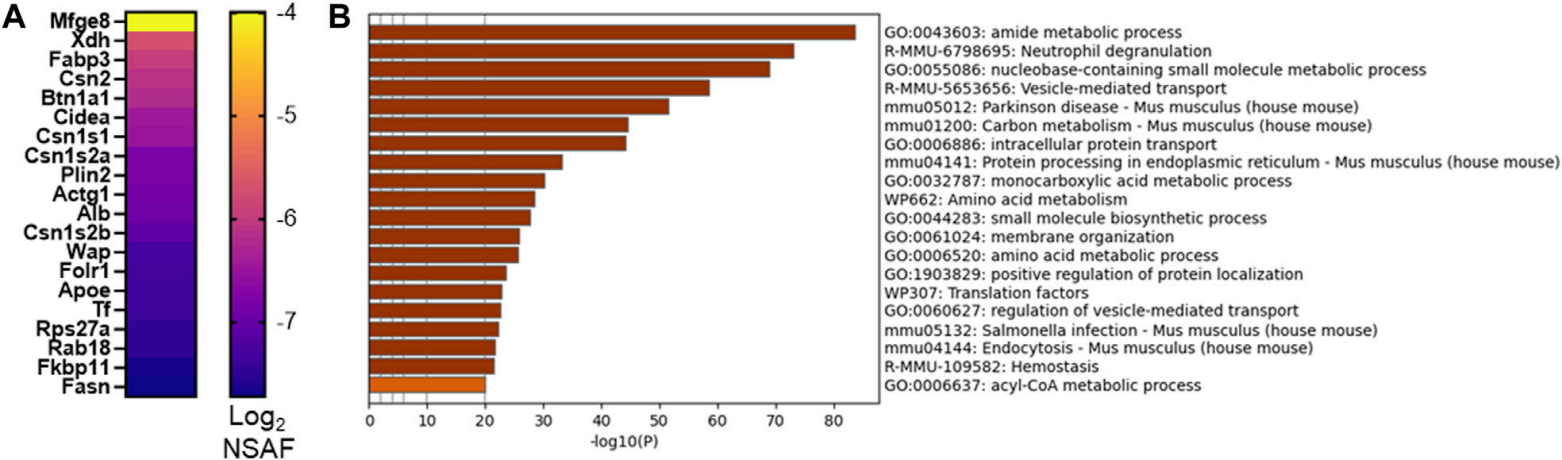
CD1 mouse MFG proteome at L10. Abundance of the top 20 MFG-associated proteins [**(A)**, Log_2_ NSAF], sorted by median. Metascape pathway enrichment analyses of MFG-associated proteins **(B)**. Top 20 pathway clusters are shown.

As we and others utilize murine models to investigate mechanisms supporting milk secretion by the mammary gland, we aimed to identify the similarities between mouse and human MFG secretion. A fuller understanding of how well the mouse mammary gland represents the human breast can inform translational research into human lactation. We therefore collected milk from lactating CD1 mouse dams and isolated MFGs. The small milk volumes available precluded us from isolating the MFGM fraction from these samples, and therefore, we were restricted to analyzing the MFG proteome, with the understanding that these proteins comprise a mix of cytoplasmic and membrane proteins. We identified 1,577 proteins present in ≥50% of samples and 1,007 proteins present in 100% of samples. A heatmap of the 20 most abundant proteins is displayed in Figure 6A, and all proteins are listed in Supplementary Table S8. We obtained similar coverage of the known CLD docking components (mostly >50%–60%; Supplementary Figure S2B). Interestingly, in contrast to human MFGs, Xdh/Xor was more highly abundant than Btn1a1 and Cidea, and these three were more highly abundant than Plin2 in murine MFG. Pathway analyses of murine MFG-associated proteins found in ≥50% of the samples identified similar pathway clusters as the human analysis, with intracellular transport, endoplasmic reticulum and ribosomal pathway clusters being well represented, with translational pathway clusters being more highly represented in the murine MFG dataset than the human MFG dataset (Figure 6B–top 20 pathway clusters, Supplementary Figure S7—top 100 pathway clusters and Supplementary Table S9—all pathways). Of the notable pathway clusters identified in the human analysis described above, we also identified neutrophil degranulation pathway clusters with murine MFG-associated proteins. Notably missing from the murine top 100 pathway cluster list was VEGFA-VEGFR2 and Rho GTPases, Miro GTPases and RHOBTB3 signaling, which were highly significant in the human pathway cluster analyses, and lipid metabolism pathway clusters were not as highly represented.

**FIGURE 7:**
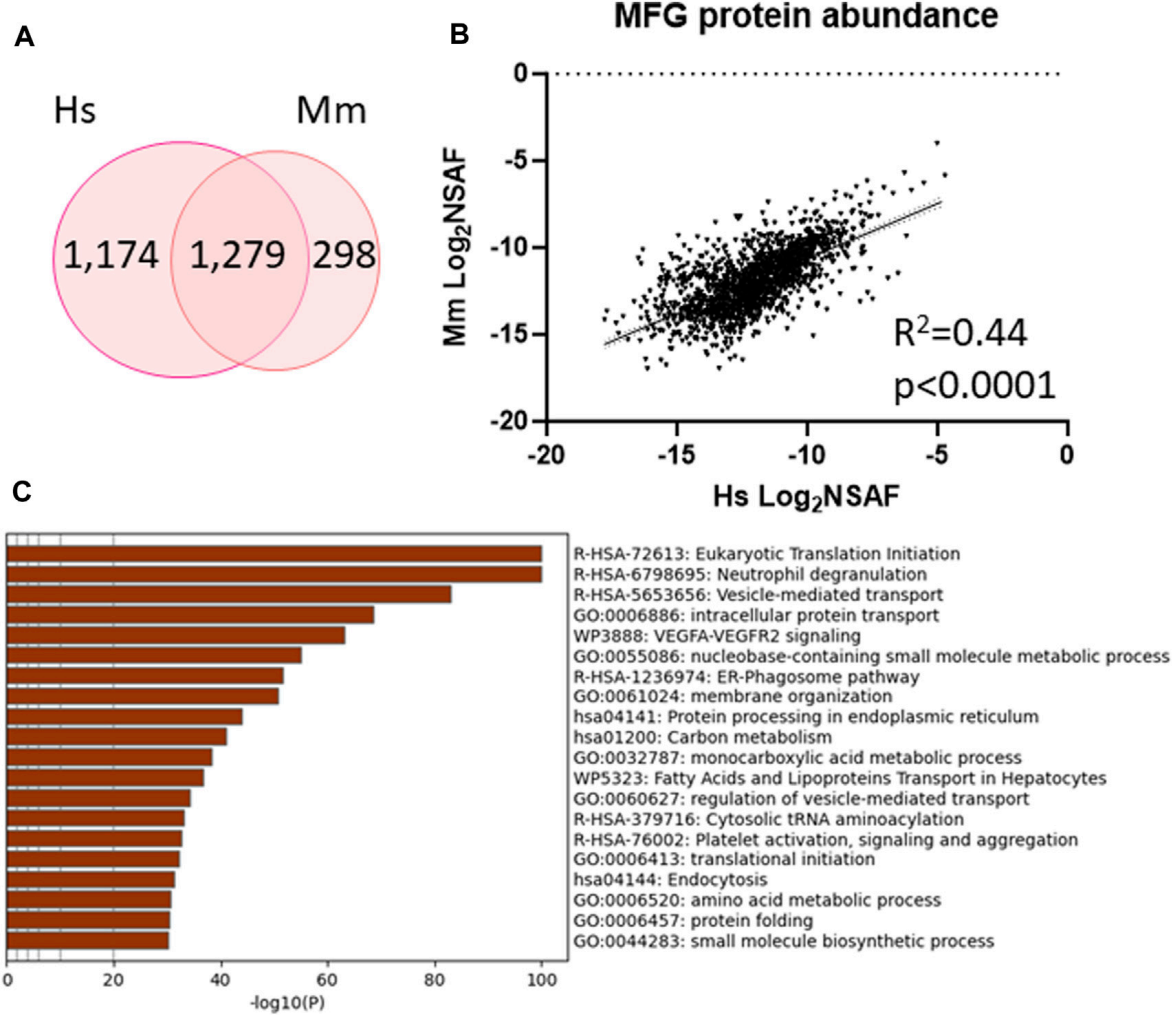
Shared protein abundance between human MFG and murine MFG. Venn diagram of shared and distinct human MFG and murine MFG-associated proteins **(A)**. Pearson correlation of abundance between proteins shared across human and murine MFGs **(B)**. Metascape pathway enrichment analyses of shared human and murine MFG-associated proteins **(C)**. Top 20 pathway clusters are shown.

To further understand the inter-species similarities, we identified proteins which were unique to either human (1,174 proteins) and mouse (298 proteins) MFG proteomes or detected in both (1,279 proteins, Figure 7A, Supplementary Table S10) and investigated how well correlated the shared proteins are (Figure 7B). We identified moderate correlation across species (*R* ^2^ = 0.44, *p* < 0.0001) and utilized this common protein list to conduct further pathway analysis (Figure 7C–top 20 pathway clusters, Supplementary Figure S8—top 100 pathway clusters and Supplementary Table S11—all pathways). Ribosome, endoplasmic reticulum, intracellular transport pathway clusters and neutrophil degranulation clusters were shared across species, indicating that the pathways identified across species largely utilize common sets of proteins within pathways rather than utilizing distinct proteins which function within the same broader pathways. Although not identified in pathway analysis of murine MFG proteins, the VEGF-VEGFR2 signaling pathway cluster was significantly overrepresented in pathway analysis of proteins common to both species.

**FIGURE 8:**
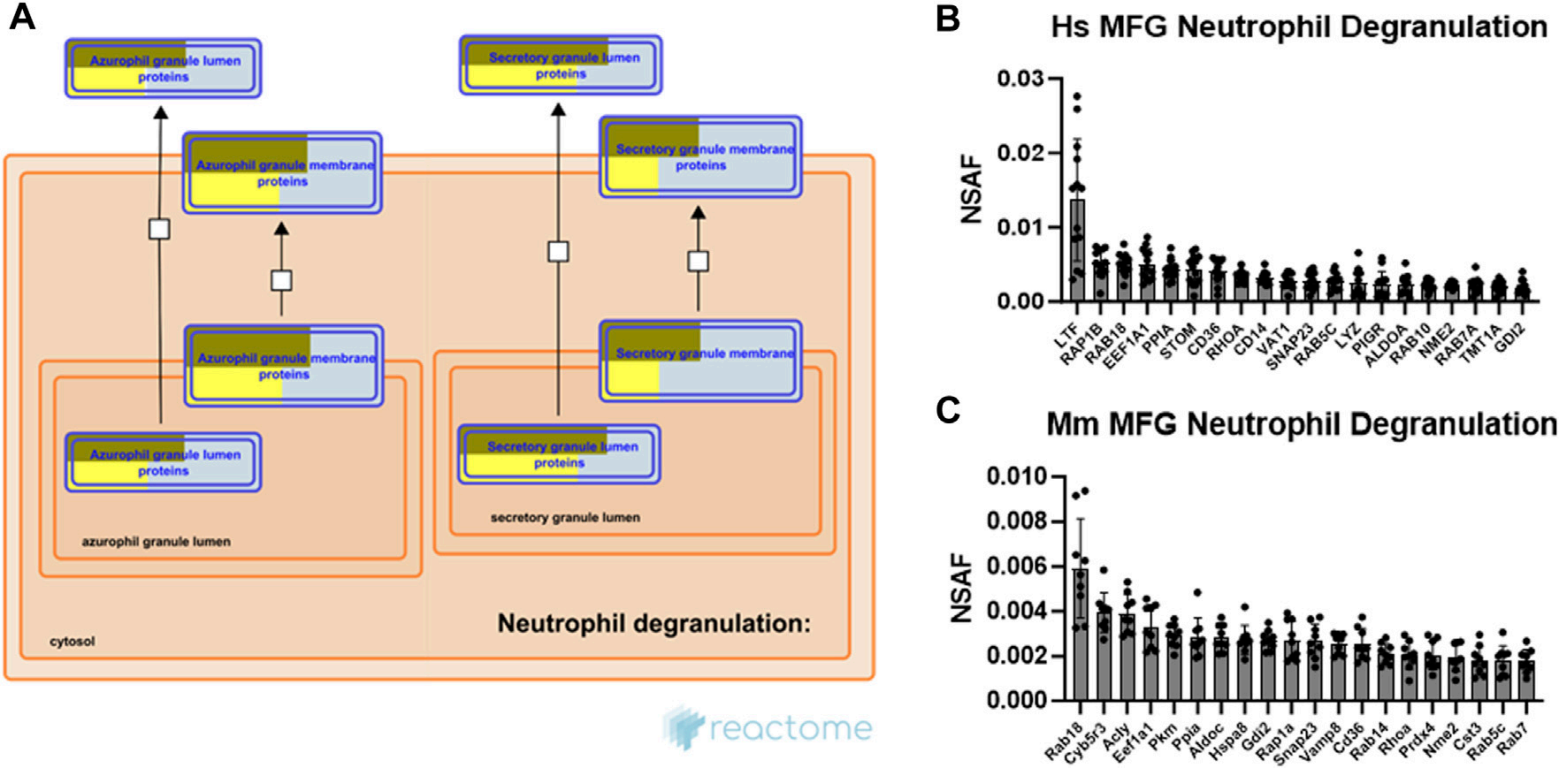
Neutrophil degranulation pathway analysis. Reactome pathway diagram with overlaid overrepresentation pathway enrichment analyses of human and mouse MFG-associated proteins in the neutrophil degranulation pathway, including only the primary (azurophil) and secretory granule entities **(A)**. Entities are re-colored (gold for human, yellow for mouse) if they were represented in the submitted data set. The top 20 most highly abundant neutrophil degranulation-related proteins from the human **(B)** and mouse **(C)** MFG proteomes, sorted by mean.

The neutrophil degranulation pathway was consistently one of the most highly significant pathways identified across species and sample types. As evidence has only recently emerged in mice that secretion of membrane-tethered CLD is regulated, at least in part, by oxytocin-mediated myoepithelial cell contraction (Masedunskas et al., 2017), the processes responsible for this newly identified stimulated form of secretion remain largely unknown. Therefore, commonalities with the carefully controlled neutrophil degranulation process may provide insight into stimulated MFG secretion mechanisms. Neutrophils contain at least 4 distinct types of preformed secretory vesicles known as granules, which are thought to form sequentially during neutrophil differentiation, and which contain distinct effector subsets (Le Cabec et al., 1996). These include primary, secondary, tertiary and secretory granules, with secondary granules containing lactoferrin, an antibacterial protein which is also highly abundant in milk (Kell et al., 2020). Pathway analysis of both human (Supplementary Figure S9A) and mouse (Supplementary Figure S9B) MFG-associated proteins identified the strongest overrepresentation of primary (azurophilic) granule membrane proteins and secretory granule lumen proteins, as illustrated by Reactome (Figure 8A).

Neutrophil degranulation entails cytoskeletal remodeling to allow for granule trafficking to the plasma membrane, granule tethering and docking, granule priming for fusion, and fusion of the granule with the plasma membrane to allow for release of its contents. Small GTPases, which are critical for cytoskeletal remodeling and vesicle trafficking, are known to regulate many of the interactions controlling these processes (Lacy, 2006). The top 20 most abundant neutrophil degranulation pathway proteins identified in human and mouse MFG-associated proteomes are shown in Figures 8B, C, respectively. Common between these top 20 proteins across species are the small GTPases RAB18, member RAS oncogene family (RAB18), which is known in other cell types to regulate lipid droplet growth (Ozeki et al., 2005; Xu et al., 2018; Deng et al., 2021), ras homolog family member A (RHOA), RAB5C, member RAS oncogene family (RAB5C), as well as the GDP dissociation inhibitor 2 (GDI2). Also highly abundant are CD36 molecule (CD36), elongation factor 1-alpha 1 (EEF1A1), peptidylprolyl isomerase A (PPIA), NME/NM23 nucleoside diphosphate kinase 2 (NME2) and synaptosome associated protein 23 (SNAP23), which has been shown to form a soluble N-ethylmaleimide sensitive factor attachment protein receptor (SNARE) complex with vesicle-associated membrane protein 8 (Vamp8) in murine mammary epithelial cells, potentially allowing secretory vesicles to contribute membrane to the MFGM in addition to the apical plasma membrane (Honvo-Houéto et al., 2016).

**FIGURE 9:**
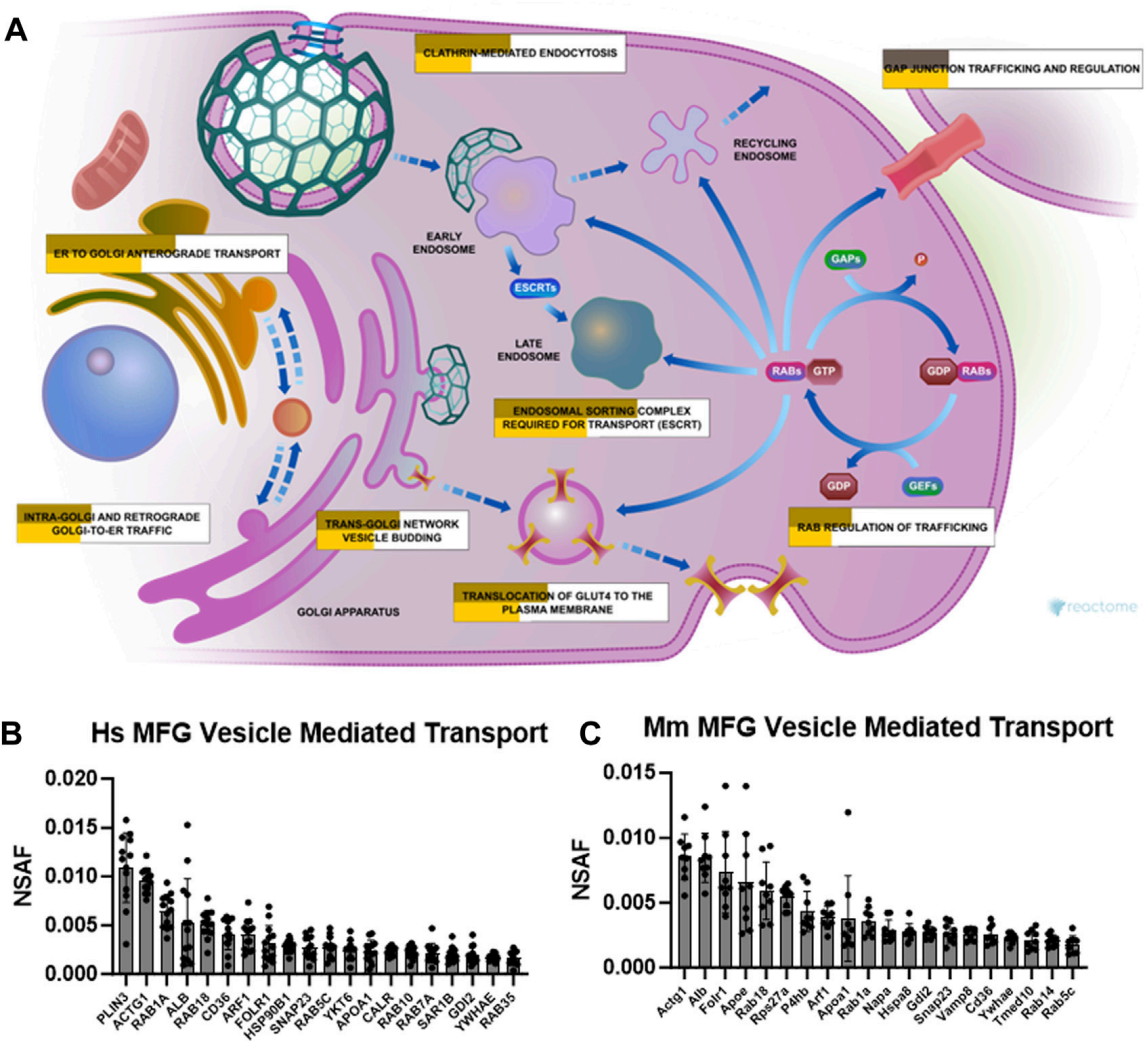
Membrane trafficking and vesicle mediated transport pathway enrichment analysis. Reactome pathway diagram with overlaid overrepresentation pathway enrichment analyses of human and mouse MFG-associated proteins in the membrane trafficking pathway **(A)**. Entities are re-colored (gold for human—upper, yellow for mouse—lower, gray if not statistically significant) if they were represented in the submitted data set. The top 20 most highly abundant vesicle mediated transport-related proteins from the human **(B)** and mouse **(C)** MFG proteomes, sorted by mean.

Related to the specialized trafficking responsible for neutrophil degranulation, we also identified vesicle mediated transport, and its subcluster membrane trafficking, as overrepresented pathway clusters in both human (Supplementary Figure S10) and mouse (Supplementary Figure S11) MFG-associated proteomes. As illustrated by Reactome, multiple pathways were represented by the membrane trafficking pathway cluster (Figure 9A) which were significantly overrepresented in our MFG datasets, including ER to Golgi anterograde transport, intra-Golgi and retrograde Golgi-to-ER traffic, trans-Golgi network vesicle budding, translocation of GLUT4 to the plasma membrane, RAB regulation of trafficking, clathrin mediated endocytosis and endosomal sorting complex required for transport (ESCRT). Gap junction trafficking and regulation was also significantly overrepresented, but only in our murine dataset. The top 20 most abundant vesicle mediated transport pathway proteins identified in human and mouse MFG-associated proteomes are shown in Figures 9B, C, respectively. Common in the top 20 most abundant pathway proteins between species are RAB18, CD36, SNAP23, RAB5C and GDI2, in addition to actin gamma 1 (ACTG1), RAB1A, member RAS oncogene family (RAB1A), albumin (ALB), ADP-ribosylation factor 1 (ARF1), folate receptor alpha (FOLR1), apolipoprotein A1 (APOA1), and tyrosine 3-monooxygenase/tryptophan 5-monooxygenase activation protein epsilon (YWHAE).

RAB18 has been shown to be involved in lipid droplet interactions with other organelles (Li et al., 2017; Zappa et al., 2017). We anticipate that the high abundance of RAB18 found in MFGs from both human and mice indicates a critical role for this GTPase in regulating MFG secretion.

**FIGURE 10:**
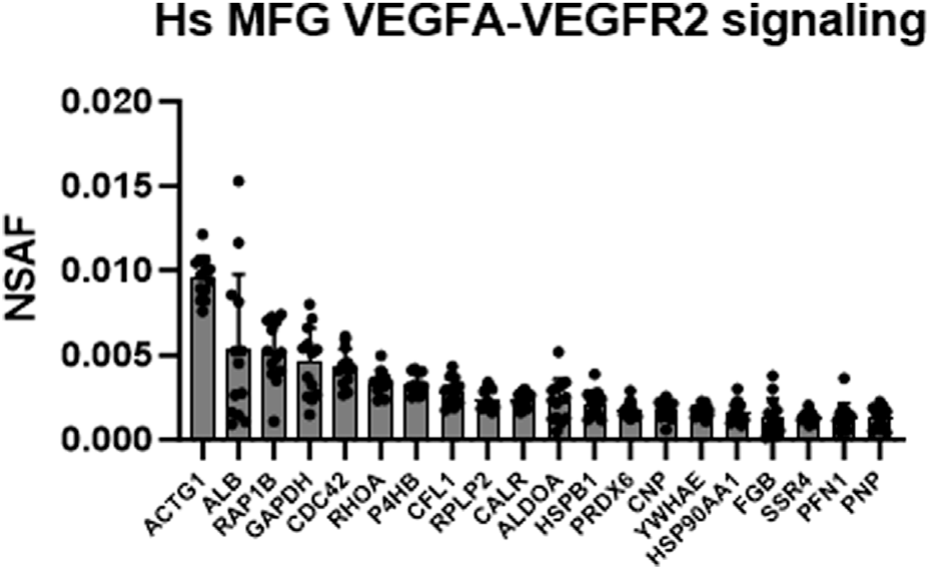
VEGFA-VEGFR2 signaling pathway analysis. Top 20 most abundant VEGF-related proteins found in the human MFG proteomes, sorted by MFG mean.

Intriguingly, the VEGFA-VEGFR2 signaling pathway cluster was identified as highly significant in Metascape pathway analyses of the human MFG proteome (Figure 5), and in the shared human and murine MFG proteome (Figure 7). We illustrate this pathway using Reactome in Supplementary Figure S12, with the top 20 most abundant proteins in this pathway identified in the human MFG displayed in Figure 10. As vascular endothelial growth factor (VEGF) is best studied for its effects on endothelial cells, potential direct effects on epithelial cells are unexpected. We did not identify any VEGF receptors in either the human MFG or MFGM proteomes, indicating that if VEGF signaling is occurring in the mammary epithelial cells which secrete MFGs, it is not occurring on the apical surface, and is likely occurring across the basal membrane. Non-catalytic region of tyrosine kinase adaptor protein 1 (NCK1) and subunits of Protein Kinase C, protein kinase C delta (PRKCD) and protein kinase C beta (PRKCB), appear to be main factors driving the identification of the VEGF signaling pathway. These signal transduction molecules are not specific to the VEGF signaling pathway, and we expect that their function in other signaling pathways active in the lactating mammary epithelial cells may be more relevant for understanding MFG synthesis and secretion.

## Discussion

MFGs are unique, nutritionally important, membrane enveloped structures that are the primary source of neonatal calories and fat-soluble vitamins, fatty acids and lipid signaling molecules implicated in neonatal development (Oftedal, 1984). We used TIMS/PASEF proteomics to quantify relative abundances of proteins in freshly isolated human MFGs and MFGM fractions and to compare mouse and human MFG proteomic profiles. Ours is the first study to comprehensively quantify the protein compositions of human MFG and their paired, mechanically isolated membrane fractions to identify proteins specifically enriched on human MFGM and sequestered in the cytoplasm. In conjunction with pathway enrichment analyses, our results advance knowledge of the composition and relative quantities of proteins in human and mouse MFG in greater detail, provide a quantitative profile of specifically enriched human MFGM proteins, and identify core cellular systems involved in forming MFGs and MFGMs.

Due to the unique apocrine mechanism of milk lipid secretion, in which portions of the apical plasma membrane, CLD, and apically targeted cellular elements, including Golgi and secretory vesicles, are released from secretory epithelial cells (McManaman, 2012; Honvo-Houéto et al., 2016; Farkaš et al., 2020), the protein composition of MFGs is predicted to be an aggregate of a select set of proteins captured from the plasma membrane, CLD, and cytoplasmic fractions of the cell during MFG secretion. Prior studies of human MFGs have largely focused on the putative MFGM fractions of these structures (Liao et al., 2011; Yang et al., 2016), and few studies have been directed at defining MFGM protein composition relative to intact MFGs and identifying the cellular systems involved in the formation of these structures. Using MS/MS analysis of human MFG proteins separated by 1D SDS-PAGE electrophoresis, Spertino et al. (2012), identified 13 of the most abundant proteins in human MFG and using LC-MS/MS of iTRAC-labeled proteins, Yang et al. (2015) identified 520 proteins from human MFG. In contrast, using TIMS/PASEF analysis, we reproducibly quantified relative abundances of 2,453 proteins in intact, freshly isolated, human MFGs and 1,577 proteins in mouse MFGs, which significantly increases the depth of knowledge about the protein composition of these structures and enhances identification of cellular pathways that contribute to their formation. For intact, freshly isolated human and mouse MFGs, we found a positive correlation in the relative abundances of 1,279 proteins that were found in common, which indicates that similar cellular systems may contribute to MFG formation in both species. Additionally, we found that the most abundant proteins in human and mouse MFG were either involved in regulating CLD-membrane interactions during MFG secretion (BTN1A1, XDH/XOR and PLIN2) (Ogg et al., 2004b; Jeong et al., 2013; Monks et al., 2016; Monks et al., 2022), were cytoplasmic or cytoplasmic secretory vesicle components MFGE8, FABP3 and caseins [casein alpha s1 (CSN1S1) and casein kappa (CSN3) in human, casein beta (Csn2), Csn1s1, casein alpha s2-like A (Csn1s2a) and casein alpha s2-like B (Csn1s2b) in mouse)], or were implicated as regulators of membrane processes and vesicle trafficking [Rab proteins (RAB18) and ACTG1 in both human and mouse]. Pathway enrichment analysis of human and mouse MFG proteomes also revealed similarities in their corresponding biological pathways. The common, most significantly enriched pathways in MFG from both species were related to vesicular transport, and membrane organization, suggesting that they may represent core cellular processes that contribute to MFG formation.

Nevertheless, we also found marked differences between human and mouse MFGs in the relative abundances of specific proteins, which demonstrates differences in their expression and/or incorporation into MFGs and in the relative contributions of certain biological processes to MFG formation. For example, LTF, which is a secreted protein found in cytoplasmic vesicles, is 5th in abundance in human MFGs versus 106th in mouse; perilipin 3 (PLIN3), which is a CLD-associated protein that is also implicated in endosomal trafficking (Bickel et al., 2009), is 7th in abundance in human MFGs versus 616th in mouse; and CIDEA, which is a CLD-associated protein in the mouse mammary gland that is implicated as a regulator of milk lipid secretion (Wang et al., 2012) is 31st in abundance in human MFG versus 6th in mouse MFGs.

We identified proteins specifically associated with human MFGMs by comparing the relative abundances of proteins found in intact MFGs from individual subjects with their relative abundances in corresponding MFGM fractions in a single LC-MS/MS run. This approach allowed us to directly compare relative protein abundances in MFGs and MFGMs from individual subjects, which eliminates the potential for inter-run variability and improves the power and quantitative rigor of MFGM protein enrichment analysis over prior studies of human MFGM proteins. These analyzed pooled samples and did not directly compare protein abundances in MFG and MFGM fractions (Liao et al., 2011; Yang et al., 2015; Yang et al., 2016; Zhang et al., 2021). Using differences in relative abundances between MFGs and isolated MFGMs, we found 699 proteins whose relative abundances were significantly enriched in MFGMs after correcting for multiple comparisons. The top 20 MFGM-enriched proteins included those demonstrated to contribute to CLD docking in mouse models of milk lipid secretion - BTN1A1 (#1), XDH/XOR (#2), PLIN2 (#3) and CIDEA (#6) (Monks et al., 2016; Monks et al., 2022) which have previously been reported to be major MFGM proteins (Reinhardt and Lippolis, 2008; Thum et al., 2022). We also found several known membrane proteins among the top 20 MFGM-enriched proteins, including the ATP binding cassette subfamily G member 2 (ABCG2, #7) that is linked to riboflavin transport in human milk (Golan and Assaraf, 2020), cytochrome B5 type A (CYB5; #8), a redox enzyme previously found on MFGM in humans and other species (Jarasch et al., 1977), toll-like receptor 2 (TLR2, #10), which has been detected on bovine MFGM (Reinhardt and Lippolis, 2008), CD59 molecule (CD59 blood group) (CD59, #11), a complement factor previously identified in human MFG (Hakulinen and Meri, 1995); and calcium and integrin binding 1 (CIB1, #17), a suppressor of integrin activation (Kim et al., 2011) and calcineurin-like EF-hand protein (CHP1, #18), a regulator of endocytosis (Janzen et al., 2018), which have not been reported to be MFGM proteins previously. In addition, lipid synthesis enzymes acetyl CoA synthetase long chain family members 1 and 4 (ACSL1 #12, ACSL4 #13) and lanosterol synthase (LSS, #15) are among the top human MFGM-enriched proteins, ACSL1 and LSS have been detected previously on bovine MFGM (Reinhardt and Lippolis, 2008). Other abundant human MFGM-enriched proteins that have been detected on MFGMs previously are lactalbumin alpha (LALBA, #4) which regulates lactose synthesis and is reported to have antimicrobial properties (Charlwood et al., 2002); eukaryotic translation elongation factor 1 alpha 1 (EEF1A1, #5), an actin-binding protein that contributes to the regulation of epithelial cell junctions (Erasmus et al., 2016); calcium binding proteins S100 calcium binding protein A1(S100A1, #9) and calnexin (CANX, #19) (Reinhardt and Lippolis, 2008; Yang et al., 2016); and syntaxin binding protein 2 (STXBP2, #22) (Reinhardt and Lippolis, 2008). We did not find that 2′,3′-cyclic nucleotide 3′-phosphodiesterase (CNP, #14), or Ribophorin 1 (RPN1, #16) had been detected on MFGMs previously.

Importantly, we found that the relative abundances of several proteins reported to be major MFGM proteins from humans or other species (Thum et al., 2022) either did not differ significantly between MFG and MFGM fractions or were significantly more abundant in the MFG fraction. The relative abundance of MFGE8, for instance, was significantly greater in human MFG compared to corresponding MFGM fractions, whereas relative abundances of FABP3, mucin1 (MUC1); and CD36 did not differ significantly between human MFG and MFGM. These results provide evidence that for some proteins previously thought to be enriched on MFGMs, their membrane association may be comparatively weak or their abundances in other MFG compartments may be comparable to their membrane abundances.

We found 201 proteins with significantly greater abundances in MFG compared to their corresponding MFGM factions. In addition to MFGE8, LTF and CSN3, which are found in secretory vesicles, we found significant increases in the abundances of several cytoplasmic proteins including enolase 1 (ENO1), RAS like proto-oncogene B (RALB), carbonic anhydrase-6 (CA6), glyceraldehyde-3-phophate dehydrogenase (GAPDH) as well as numerous ribosomal proteins (RPS7, RPS17, RPS15A and RPS15) in MFG relative to MFGM fractions. Notably, pathway analyses of MFG-associated proteins showed greater enrichment of ribosomal proteins in mouse than in human, although these proteins were evident in the human dataset of MFG-enriched, or cytoplasmic, proteins, suggesting the possibility that more or larger cytoplasmic components are captured upon MFG secretion in mice than in humans. Collectively these data are consistent with the apocrine mechanism of lipid secretion, which is proposed to capture soluble and vesicular fractions of the cytoplasm in addition to CLD. However, mouse data have shown that apocrine lipid secretion is facilitated by, but does not require, contact between CLD and the apical plasma membrane (Monks et al., 2016; Monks et al., 2022). Thus, variations in the extent of CLD docking or in maternal physiological processes provide opportunities for variable combinations of plasma, vesicular and organellar membranes, and cytoplasmic components to be included in secreted MFGs.

By PCA, we found differences in relative abundances of human MFGM-associated proteins between transitional vs. mature lactation timepoints that was driven primarily by the significantly increased abundance of BTN1A1 in MFGMs over time. Although previous human and bovine studies have reported that the expression of protein mediators of CLD-membrane interactions including BTN1A1, XDH/XOR, PLIN2 and CIDEA increase between colostral and mature phases of lactation (Reinhardt and Lippolis, 2008; Yang et al., 2016), we did not detect significant differences in the relative abundances of XDH/XOR, PLIN2 or CIDEA in MFGM fractions from transitional and mature milk in our cohort, which suggests that molecular complexes involved in docking CLDs to the apical membrane are largely established in humans by the 2nd week of lactation.

Pathway analysis of the proteins enriched in human MFGMs identified lipid biosynthesis and localization as among the most significantly enriched pathways. In addition, several significantly enriched pathways related to MFG proteins were also found to be significantly enriched in analysis of MFGM-enriched proteins, including vesicle transport and membrane organization. These findings are consistent with the proposal that vesicular compartments contribute to MFGM formation (Wooding, 1973; Wooding, 2023). We also identified significant enrichment of ER-associated pathways in human MFGM-enriched proteins, including N-linked glycosylation. The enrichment of these pathways in the human MFGM is consistent with studies in mice proposing that ER proteins contribute to MFGM formation (Wu et al., 2000; Honvo-Houéto et al., 2016). This ER-related pathway is related to protein folding, which suggests that discrete ER elements required for correct protein folding and/or protein quality control may be specifically directed to apocrine lipid secretion. Consistent with this mechanism, our proteomics data show that several proteins implicated in tethering the ER to the plasma membrane (Li et al., 2021) are enriched in isolated human MFGM, including VAMP associated protein A (VAPA, #68), extended synaptotagmin proteins ESYT1 (# 199) and ESYT2 (# 31) and oxysterol-binding protein like proteins OSBPL2 (# 233), OSBPL1A (# 318) and OSBPL8 (# 391).

We unexpectedly identified the neutrophil degranulation pathway to be highly enriched for both human MFG and MFGM and mouse MFG protein lists. We anticipate that there may be multiple reasons for this signal. We speculate that the mammary gland has borrowed some of the molecular processes utilized by neutrophil degranulation for stimulated secretion mechanisms and repurposed them for MFG secretion. However, this signal may also be related to known immune functions of the MFG (Brink and Lonnerdal, 2020). Our pathway analyses suggest that in addition to lipid transfer, the structure of MFGs may convey neutrophil-related immunological protection for infants, beyond the previously recognized bactericidal effects of XDH/XOR and LTF. We and others (Lemay et al., 2013) have found that milk secretion pathways are poorly annotated in the databases used for pathway analysis, and improved annotation of these pathways may support research into mechanisms which alter human milk production and/or composition.

Recently, the *Eunice Kennedy Shriver* National Institute of Child Health and Human Development (NICHD) of the National Institutes of Health (NIH) initiated the “Breastmilk Ecology: Genesis of Infant Nutrition (BEGIN)” Project (Raiten et al., 2023), which convened leaders in the field of lactation science to “explore factors influencing the synthesis, composition, and best use of human milk.” The BEGIN working groups conceptualized new approaches toward understanding human milk as a complex biological system (Donovan et al., 2023; Krebs et al., 2023; Neville et al., 2023; Nommsen-Rivers et al., 2023; Smilowitz et al., 2023). Indeed, milk fat secretion is thought to be influenced by myriad factors within the context of the mother-milk-infant triad. Milk fat composition has been shown to be linked to mother’s diet and metabolism (Bravi et al., 2016; Daniel et al., 2021), to vary across a single feed and the circadian cycle (Neville et al., 1984; Khan et al., 2013). Maternal diet and metabolism have been shown to affect MFG size, and therefore protein content as MFGM surface area is altered (Argov-Argaman, 2019). Additionally, infant-related factors, such as frequency of feeding, are expected to affect the amount of plasma membrane included in MFGs (Masedunskas et al., 2017; Mather et al., 2019). As a secondary analysis, with limited samples, our study was not designed to analyze MFG and MFGM in context with other elements of the mother-milk-infant triad. Rather, we have contributed a method which can be incorporated into the framework of larger future studies. In particular, we propose that freshly collected MFGs should be washed of other milk components prior to freezing, and that mechanical disruption and centrifugation of MFGMs may be utilized when aiming to investigate membrane-specific functions of the MFG, so as to minimize contributions of cytoplasmic proteins. As many different factors, including time postpartum, maternal diet, metabolism, circadian rhythm, drug usage and frequency of milk removal are likely to affect proteins present on MFGs and MFGMs, future research into these factors and their effects on the MFG and MFGM proteome will provide crucial information related to regulation of the core systems driving MFG synthesis and secretion. Here, we report the presence of multiple vesicular transport pathway pathways on the MFGM. These are responsible for secretion of other milk components, and we posit that these interactions contribute to a “system within a system” by which the mammary epithelial cell regulates the balance of milk components within a narrow range of values, potentially by pairing the balance of membrane lost in MFG secretion with membranes from secretory vesicles containing lactose or milk proteins. Our findings underscore the importance of understanding the contributions of MFGs in the larger context of milk composition and infant health and immunity.

In summary, we have used TIMS/PASEF proteomics to identify and define relative abundances of proteins in human MFG and MFGM and mouse MFG in greater detail than previously known. Coupled with bioinformatic pathway analyses, our results provide new information about the protein compositions of human and mouse MFGs and the cellular processes that contribute to their formation. By comparing relative abundances of human MFG and MFGM proteins we were able to identify a set of proteins that are specifically enriched on human MFGMs. Collectively these data provided new insight into the protein compositions of human MFGs and MFGMs and the cellular processes involved in their formation, which we speculate will help to define the importance of these unique structures in infant nutrition.

## Data Availability

The original contributions presented in the study are publicly available. These data can be found here: Center for Computational Mass Spectrometry (CCMS), Mass Spectrometry Interactive Virtual Environment (MassIVE), https://massive.ucsd.edu/ProteoSAFe/static/massive.jsp, MSV000092892 and MSV000092915.

## Supplementary material

SUPPLEMENTARY FIGURE S1 Multiple non-parametric (Mann-Whitney) unpaired t-tests of MFGM-associated proteins between early (2 weeks) and mature (2–5 months) lactation timepoints with FDR correction for multiple comparisons (5%).

SUPPLEMENTARY FIGURE S2 Percent coverage of top 20 human MFG- (dark gray bars) and MFGM-associated (light gray bars) proteins **(A)**. Multiple paired t-tests, adjusted for multiple testing with the Holm-Šídák method. **p* < 0.05. Percent coverage of top 20 CD1 mouse MFG-associated proteins **(B)**.

SUPPLEMENTARY FIGURE S3 Metascape pathway enrichment analysis of human MFGM-enriched proteins, calculated by difference. Top 100 pathway clusters are shown, using a threshold of proteins found in ≥50% **(A)** or 100% **(B)** of one or both sample types.

SUPPLEMENTARY FIGURE S4 Metascape pathway enrichment analysis of all human MFGM-associated proteins Top 100 pathway clusters are shown, using a threshold of proteins found in ≥50% **(A)** or 100% **(B)** of MFGM replicates.

SUPPLEMENTARY FIGURE S5 Metascape pathway enrichment analysis of all human MFG-associated proteins. Top 100 pathway clusters are shown, using a threshold of proteins found in ≥50% **(A)** or 100% **(B)** of MFG replicates.

SUPPLEMENTARY FIGURE S6 Metascape pathway enrichment analysis of all human MFG-enriched (cytoplasmic) proteins. Top 100 pathway clusters are shown, using a threshold of proteins found in ≥50% **(A)** or 100% **(B)** of one or both sample types.

SUPPLEMENTARY FIGURE S7 Metascape pathway enrichment analyses of mouse MFG-associated proteins. Top 100 pathway clusters are shown, using a threshold of proteins found in ≥50% **(A)** or 100% **(B)** of MFG replicates.

SUPPLEMENTARY FIGURE S8 Metascape pathway enrichment analyses of MFG-associated proteins common to human and mouse. Top 100 pathway clusters are shown, using a threshold of proteins found in ≥50% **(A)** or 100% **(B)** of MFG replicates.

SUPPLEMENTARY FIGURE S9 Neutrophil degranulation pathway analysis. Reactome pathway diagrams with overlaid overrepresentation pathway enrichment analyses of human **(A)** and mouse **(B)** MFG-associated proteins in the neutrophil degranulation pathway. Entities are re-colored (gold for human, yellow for mouse) if they were represented in the submitted data set.

SUPPLEMENTARY FIGURE S10 Reactome pathway diagrams with overlaid overrepresentation pathway enrichment analyses of human MFG-associated proteins in the vesicle mediated transport pathway. Entities are re-colored (yellow if statistically significant, gray if not statistically significant) if they were represented in the submitted data set.

SUPPLEMENTARY FIGURE S11 Reactome pathway diagrams with overlaid overrepresentation pathway enrichment analyses of mouse MFG-associated proteins in the vesicle mediated transport pathway. Entities are re-colored (yellow if statistically significant, gray if not statistically significant) if they were represented in the submitted data set.

SUPPLEMENTARY FIGURE S12 VEGFA-VEGFR2 signaling pathway analysis. Reactome pathway diagram with overlaid overrepresentation pathway enrichment analyses of human MFG-associated proteins in the signaling by VEGF pathway. Entities are re-colored (gold) if they were represented in the submitted data set.

SUPPLEMENTARY TABLE S1 MFG and MFGM-associated proteins identified by LC-MS/MS. Abundance of all human MFG- and MFGM-associated proteins identified with UniProt Accession Numbers, sorted by means of MFG and MFGM values, combined. Average and standard deviations of peptide count and % coverage obtained are provided.

SUPPLEMENTARY TABLE S2 Differentially abundant proteins by absolute difference. Multiple paired t-tests of MFG- vs. MFGM-associated proteins, calculated by absolute difference (MFGM-MFG).

SUPPLEMENTARY TABLE S3 Differentially abundant proteins by MFG-normalized difference. Multiple paired t-tests of MFG- vs MFGM-associated proteins, calculated by differences in Log2-transformed, MFG-normalized values, [Log2(MFGM/AvgMFG)-Log2(MFG/AvgMFG)].

SUPPLEMENTARY TABLE S4 MFGM-enriched protein pathway enrichment analysis. The terms within each cluster displayed in Figure 3A and Supplementary Figure S3 are listed.

SUPPLEMENTARY TABLE S5 MFGM-associated protein pathway enrichment analysis. The terms within each cluster displayed in Figure 3C and Supplementary Figure S4 are listed.

SUPPLEMENTARY TABLE S6 MFG protein pathway enrichment analysis. The terms within each cluster displayed in Figure 4A and Supplementary Figure S5 are listed.

SUPPLEMENTARY TABLE S7 MFG protein pathway enrichment analysis. The terms within each cluster displayed in Figure 4B and Supplementary Figure S6 are listed.

SUPPLEMENTARY TABLE S8 CD1 mouse MFG proteome at L10 identified by LC-MS/MS. Abundance of all human MFG-associated proteins identified, sorted by MFG medians (normality not assumed with *n* = 9 samples).

SUPPLEMENTARY TABLE S9 Murine MFG protein pathway enrichment analysis. The terms within each cluster displayed in Figure 5A and Supplementary Figure S7 are listed.

SUPPLEMENTARY TABLE S10 Cross-species MFG similarity. Shared and distinct human MFG and murine MFG-associated proteins are listed. Means are listed for murine samples, despite not assuming normality, to provide metrics comparable to human samples.

SUPPLEMENTARY TABLE S11 Cross species MFG protein pathway enrichment analysis. The terms within each cluster displayed in Figure 6C and Supplementary Figure S8 are listed.

## References

[B1] Argov-Argaman N. (2019). Symposium review: milk fat globule size: practical implications and metabolic regulation. J. Dairy Sci. 102 (3), 2783–2795. 10.3168/jds.2018-15240 30639008

[B2] Ballard O. Morrow A. L. (2013). Human milk composition: nutrients and bioactive factors. Pediatr. Clin. North Am. 60 (1), 49–74. 10.1016/j.pcl.2012.10.002 23178060 PMC3586783

[B3] Ballard Mimmi S. Zhu A. Iwai N. Stensrud M. Mapps A. Postiglione Maira P. (2015). Mammary stem cell self-renewal is regulated by slit2/robo1 signaling through SNAI1 and mINSC. Cell Rep. 13 (2), 290–301. 10.1016/j.celrep.2015.09.006 26440891 PMC4606466

[B4] Barneda D. Planas-Iglesias J. Gaspar M. L. Mohammadyani D. Prasannan S. Dormann D. (2015). The brown adipocyte protein CIDEA promotes lipid droplet fusion via a phosphatidic acid-binding amphipathic helix. eLife 4, e07485. 10.7554/eLife.07485 26609809 PMC4755750

[B5] Beigneux A. P. Vergnes L. Qiao X. Quatela S. Davis R. Watkins S. M. (2006). Agpat6—a novel lipid biosynthetic gene required for triacylglycerol production in mammary epithelium. J. Lipid Res. 47 (4), 734–744. 10.1194/jlr.M500556-JLR200 16449762 PMC3196597

[B6] Bickel P. E. Tansey J. T. Welte M. A. (2009). PAT proteins, an ancient family of lipid droplet proteins that regulate cellular lipid stores. Biochimica Biophysica Acta (BBA) - Mol. Cell Biol. Lipids 1791 (6), 419–440. 10.1016/j.bbalip.2009.04.002 PMC2782626 19375517

[B7] Bravi F. Wiens F. Decarli A. Dal Pont A. Agostoni C. Ferraroni M. (2016). Impact of maternal nutrition on breast-milk composition: a systematic review. Am. J. Clin. Nutr. 104 (3), 646–662. 10.3945/ajcn.115.120881 27534637

[B8] Brink L. R. Lonnerdal B. (2020). Milk fat globule membrane: the role of its various components in infant health and development. J. Nutr. Biochem. 85, 108465. 10.1016/j.jnutbio.2020.108465 32758540

[B9] Cavaletto M. Giuffrida M. G. Fortunato D. Gardano L. Dellavalle G. Napolitano L. (2002). A proteomic approach to evaluate the butyrophilin gene family expression in human milk fat globule membrane. PROTEOMICS 2 (7), 850–856. 10.1002/1615-9861(200207)2:7<850::AID-PROT850>3.0.CO;2-C 12124930

[B10] Cazares O. Chatterjee S. Lee P. Strietzel C. Bubolz J. W. Harburg G. (2021). Alveolar progenitor differentiation and lactation depends on paracrine inhibition of Notch via ROBO1/CTNNB1/JAG1. Development 148 (21), dev199940. 10.1242/dev.199940 34758082 PMC8627605

[B11] Charlwood J. Hanrahan S. Tyldesley R. Langridge J. Dwek M. Camilleri P. (2002). Use of proteomic methodology for the characterization of human milk fat globular membrane proteins. Anal. Biochem. 301 (2), 314–324. 10.1006/abio.2001.5498 11814302

[B12] Christian P. Smith E. R. Lee S. E. Vargas A. J. Bremer A. A. Raiten D. J. (2021). The need to study human milk as a biological system. Am. J. Clin. Nutr. 113 (5), 1063–1072. 10.1093/ajcn/nqab075 33831952 PMC8106761

[B13] Daniel A. I. Shama S. Ismail S. Bourdon C. Kiss A. Mwangome M. (2021). Maternal BMI is positively associated with human milk fat: a systematic review and meta-regression analysis. Am. J. Clin. Nutr. 113 (4), 1009–1022. 10.1093/ajcn/nqaa410 33675341 PMC8023843

[B14] Deng Y. Zhou C. Mirza A. H. Bamigbade A. T. Zhang S. Xu S. (2021). Rab18 binds PLIN2 and ACSL3 to mediate lipid droplet dynamics. Biochimica Biophysica Acta (BBA) - Mol. Cell Biol. Lipids 1866 (7), 158923. 10.1016/j.bbalip.2021.158923 33713834

[B15] Donovan S. M. Aghaeepour N. Andres A. Azad M. B. Becker M. Carlson S. E. (2023). Evidence for human milk as a biological system and recommendations for study design—a report from “Breastmilk Ecology: Genesis of Infant Nutrition (BEGIN)” Working Group 4. Am. J. Clin. Nutr. 117, S61–S86. 10.1016/j.ajcnut.2022.12.021 37173061 PMC10356565

[B16] Dylewski D. P. Dapper C. H. Valivullah H. M. Deeney J. T. Keenan T. W. (1984). Morphological and biochemical characterization of possible intracellular precursors of milk lipid globules. Eur. J. Cell Biol. 35 (1), 99–111. 6489364

[B17] Erasmus J. C. Bruche S. Pizarro L. Maimari N. Poggioli T. Tomlinson C. (2016). Defining functional interactions during biogenesis of epithelial junctions. Nat. Commun. 7 (1), 13542. 10.1038/ncomms13542 27922008 PMC5150262

[B18] Fabregat A. Sidiropoulos K. Viteri G. Marin-Garcia P. Ping P. Stein L. (2018). Reactome diagram viewer: data structures and strategies to boost performance. Bioinforma. Oxf. Engl. 34 (7), 1208–1214. 10.1093/bioinformatics/btx752 PMC6030826 29186351

[B19] Farkaš R. Beňo M. Beňová-Liszeková D. Raška I. Raška O. (2020). A new look at transudation: the apocrine connection. Physiol. Res. 69 (2), 227–244. 10.33549/physiolres.934229 32199009 PMC8565947

[B23] Fortunato D. Giuffrida M. G. Cavaletto M. Garoffo L. P. Dellavalle G. Napolitano L. (2003). Structural proteome of human colostral fat globule membrane proteins. PROTEOMICS 3 (6), 897–905. 10.1002/pmic.200300367 12833513

[B24] Golan Y. Assaraf Y. G. (2020). Genetic and physiological factors affecting human milk production and composition. Nutrients 12 (5), 1500. 10.3390/nu12051500 32455695 PMC7284811

[B25] Görs S. Kucia M. Langhammer M. Junghans P. Metges C. C. (2009). Technical note: milk composition in mice—methodological aspects and effects of mouse strain and lactation day. J. Dairy Sci. 92 (2), 632–637. 10.3168/jds.2008-1563 19164675

[B26] Hakulinen J. Meri S. (1995). Shedding and enrichment of the glycolipid-anchored complement lysis inhibitor protectin (CD59) into milk fat globules. Immunology 85 (3), 495–501. 7558140 PMC1383925

[B27] Harburg G. Compton J. Liu W. Iwai N. Zada S. Marlow R. (2014). SLIT/ROBO2 signaling promotes mammary stem cell senescence by inhibiting wnt signaling. Stem Cell Rep. 3 (3), 385–393. 10.1016/j.stemcr.2014.07.007 PMC4266005 25241737

[B28] Hernandez T. L. Farabi S. S. Fosdick B. K. Hirsch N. Dunn E. Z. Rolloff K. (2023). Randomization to a provided higher-complex-carbohydrate versus conventional diet in gestational diabetes mellitus results in similar newborn adiposity. Diabetes Care 46 (11), 1931–1940. 10.2337/dc23-0617 37643311 PMC10620537

[B29] Hernandez T. L. Van Pelt R. E. Anderson M. A. Daniels L. J. West N. A. Donahoo W. T. (2014). A higher-complex carbohydrate diet in gestational diabetes mellitus achieves glucose targets and lowers postprandial lipids: a randomized crossover study. Diabetes Care 37 (5), 1254–1262. 10.2337/dc13-2411 24595632 PMC3994935

[B30] Hernandez T. L. Van Pelt R. E. Anderson M. A. Reece M. S. Reynolds R. M. de la Houssaye B. A. (2016). Women with gestational diabetes mellitus randomized to a higher–complex carbohydrate/low-fat diet manifest lower adipose tissue insulin resistance, inflammation, glucose, and free fatty acids: a pilot study. Diabetes Care 39 (1), 39–42. 10.2337/dc15-0515 26223240 PMC4686845

[B31] Honvo-Houéto E. Henry C. Chat S. Layani S. Truchet S. (2016). The endoplasmic reticulum and casein-containing vesicles contribute to milk fat globule membrane. Mol. Biol. Cell 27 (19), 2946–2964. 10.1091/mbc.E16-06-0364 27535430 PMC5042581

[B32] Huang D. W. Sherman B. T. Lempicki R. A. (2009). Systematic and integrative analysis of large gene lists using DAVID bioinformatics resources. Nat. Protoc. 4 (1), 44–57. 10.1038/nprot.2008.211 19131956

[B33] Ishii T. Aoki N. Noda A. Adachi T. Nakamura R. Matsuda T. (1995). Carboxy-terminal cytoplasmic domain of mouse butyrophilin specifically associates with a 150-kDa protein of mammary epithelial cells and milk fat globule membrane. Biochimica Biophysica Acta (BBA) - General Subj. 1245 (3), 285–292. 10.1016/0304-4165(95)00102-6 8541302

[B34] Janzen E. Mendoza-Ferreira N. Hosseinibarkooie S. Schneider S. Hupperich K. Tschanz T. (2018). CHP1 reduction ameliorates spinal muscular atrophy pathology by restoring calcineurin activity and endocytosis. Brain 141 (8), 2343–2361. 10.1093/brain/awy167 29961886 PMC6061875

[B35] Jarasch E. D. Bruder G. Keenan T. W. Franke W. W. (1977). Redox constituents in milk fat globule membranes and rough endoplasmic reticulum from lactating mammary gland. J. Cell Biol. 73 (1), 223–241. 10.1083/jcb.73.1.223 856833 PMC2109886

[B36] Jeong J. Kadegowda A. K. G. Meyer T. J. Jenkins L. M. Dinan J. C. Wysolmerski J. J. (2021). The butyrophilin 1a1 knock-out mouse revisited: ablation of Btn1a1 leads to concurrent cell death and renewal in the mammary epithelium during lactation. FASEB BioAdvances 3 (12), 971–997. 10.1096/fba.2021-00059 34938960 PMC8664049

[B37] Jeong J. Lisinski I. Kadegowda A. K. G. Shin H. Wooding F. B. P. Daniels B. R. (2013). A test of current models for the mechanism of milk-lipid droplet secretion. Traffic 14 (9), 974–986. 10.1111/tra.12087 23738536 PMC4524534

[B38] Jeong J. Rao A. U. Xu J. Ogg S. L. Hathout Y. Fenselau C. (2009). The PRY/SPRY/B30.2 Domain of Butyrophilin 1A1 (BTN1A1) Binds to Xanthine Oxidoreductase: implications for the function of btn1a1 in the mammary gland and other tissues. J. Biol. Chem. 284 (33), 22444–22456. 10.1074/jbc.M109.020446 19531472 PMC2755966

[B39] Juvarajah T. Wan-Ibrahim W. I. Ashrafzadeh A. Othman S. Hashim O. H. Fung S. Y. (2018). Human milk fat globule membrane contains hundreds of abundantly expressed and nutritionally beneficial proteins that are generally lacking in caprine milk. Breastfeed. Med. 13 (9), 631–637. 10.1089/bfm.2018.0057 30362820

[B40] Kassan A. Herms A. Fernández-Vidal A. Bosch M. Schieber N. L. Reddy B. J. N. (2013). Acyl-CoA synthetase 3 promotes lipid droplet biogenesis in ER microdomains. J. Cell Biol. 203 (6), 985–1001. 10.1083/jcb.201305142 24368806 PMC3871434

[B41] Keenan T. W. Patton S. (1995). “The structure of milk: implications for sampling and storage: A. The milk lipid globule membrane,” in Handbook of milk composition. Editor Jensen R. G. (San Diego: Academic Press), 5–50.

[B42] Kell D. B. Heyden E. L. Pretorius E. (2020). The Biology of lactoferrin, an iron-binding protein that can help defend against viruses and bacteria. Front. Immunol. 11, 1221. 10.3389/fimmu.2020.01221 32574271 PMC7271924

[B43] Khan S. Hepworth A. R. Prime D. K. Lai C. T. Trengove N. J. Hartmann P. E. (2013). Variation in fat, lactose, and protein composition in breast milk over 24 hours:associations with infant feeding patterns. J. Hum. Lactation 29 (1), 81–89. 10.1177/0890334412448841 22797414

[B44] Kim C. Ye F. Ginsberg M. H. (2011). Regulation of integrin activation. Annu. Rev. Cell Dev. Biol. 27 (1), 321–345. 10.1146/annurev-cellbio-100109-104104 21663444

[B45] Krebs N. F. Belfort M. B. Meier P. P. Mennella J. A. O’Connor D. L. Taylor S. N. (2023). Infant factors that impact the ecology of human milk secretion and composition—a report from “Breastmilk Ecology: Genesis of Infant Nutrition (BEGIN)” Working Group 3. Am. J. Clin. Nutr. 117, S43–S60. 10.1016/j.ajcnut.2023.01.021 37173060 PMC10356564

[B46] Kurosumi K. Kobayashi Y. Baba N. (1968). The fine structure of mammary glands of lactating rats, with special reference to the apocrine secretion. Exp. Cell Res. 50 (1), 177–192. 10.1016/0014-4827(68)90406-0 5689903

[B47] Lacy P. (2006). Mechanisms of degranulation in neutrophils. Allergy, Asthma and Clin. Immunol. 2 (3), 98–108. 10.1186/1710-1492-2-3-98 20525154 PMC2876182

[B48] Le Cabec V. Cowland J. B. Calafat J. Borregaard N. (1996). Targeting of proteins to granule subsets is determined by timing and not by sorting: the specific granule protein NGAL is localized to azurophil granules when expressed in HL-60 cells. Proc. Natl. Acad. Sci. 93 (13), 6454–6457. 10.1073/pnas.93.13.6454 8692836 PMC39044

[B49] Lemay D. G. Ballard O. A. Hughes M. A. Morrow A. L. Horseman N. D. Nommsen-Rivers L. A. (2013). RNA sequencing of the human milk fat layer transcriptome reveals distinct gene expression profiles at three stages of lactation. PLOS ONE 8 (7), e67531. 10.1371/journal.pone.0067531 23861770 PMC3702532

[B50] Li C. Luo X. Zhao S. Siu G. K. Liang Y. Chan H. C. (2017). COPI–TRAPPII activates Rab18 and regulates its lipid droplet association. EMBO J. 36 (4), 441–457. 10.15252/embj.201694866 28003315 PMC5694949

[B51] Li C. Qian T. He R. Wan C. Liu Y. Yu H. (2021). Endoplasmic reticulum–plasma membrane contact sites: regulators, mechanisms, and physiological functions. Front. Cell Dev. Biol. 9, 627700. 10.3389/fcell.2021.627700 33614657 PMC7889955

[B52] Liao Y. Alvarado R. Phinney B. Lönnerdal B. (2011). Proteomic characterization of human milk fat globule membrane proteins during a 12 Month lactation period. J. Proteome Res. 10 (8), 3530–3541. 10.1021/pr200149t 21714549

[B53] Listenberger L. L. Ostermeyer-Fay A. G. Goldberg E. B. Brown W. J. Brown D. A. (2007). Adipocyte differentiation-related protein reduces the lipid droplet association of adipose triglyceride lipase and slows triacylglycerol turnover. J. Lipid Res. 48 (12), 2751–2761. 10.1194/jlr.M700359-JLR200 17872589

[B54] Lu J. Wang X. Zhang W. Liu L. Pang X. Zhang S. (2016). Comparative proteomics of milk fat globule membrane in different species reveals variations in lactation and nutrition. Food Chem. 196, 665–672. 10.1016/j.foodchem.2015.10.005 26593540

[B55] Macias H. Moran A. Samara Y. Moreno M. Compton Jennifer E. Harburg G. (2011). SLIT/ROBO1 signaling suppresses mammary branching morphogenesis by limiting basal cell number. Dev. Cell 20 (6), 827–840. 10.1016/j.devcel.2011.05.012 21664580 PMC3129866

[B56] Marlow R. Binnewies M. Sorensen L. K. Monica S. D. Strickland P. Forsberg E. C. (2010). Vascular Robo4 restricts proangiogenic VEGF signaling in breast. Proc. Natl. Acad. Sci. 107 (23), 10520–10525. 10.1073/pnas.1001896107 20498081 PMC2890778

[B57] Martin Carli J. F. Trahan G. D. Jones K. L. Hirsch N. Rolloff K. P. Dunn E. Z. (2020). Single cell RNA sequencing of human milk-derived cells reveals sub-populations of mammary epithelial cells with molecular signatures of progenitor and mature States: a novel, non-invasive framework for investigating human lactation Physiology. J. Mammary Gl. Biol. Neoplasia 25, 367–387. 10.1007/s10911-020-09466-z PMC8016415 33216249

[B58] Masedunskas A. Chen Y. Stussman R. Weigert R. Mather I. H. (2017). Kinetics of milk lipid droplet transport, growth, and secretion revealed by intravital imaging: lipid droplet release is intermittently stimulated by oxytocin. Mol. Biol. Cell 28 (7), 935–946. 10.1091/mbc.E16-11-0776 28179456 PMC5385942

[B59] Mather I. H. (2000). A review and proposed nomenclature for major proteins of the milk-fat globule membrane. J. Dairy Sci. 83 (2), 203–247. 10.3168/jds.S0022-0302(00)74870-3 10714856

[B60] Mather I. H. Jack L. J. W. Madara P. J. Johnson V. G. (2001). The distribution of MUC1, an apical membrane glycoprotein, in mammary epithelial cells at the resolution of the electron microscope: implications for the mechanism of milk secretion. Cell Tissue Res. 304 (1), 91–101. 10.1007/s004410100351 11383890

[B61] Mather I. H. Keenan T. W. (1998). Origin and secretion of milk lipids. J. Mammary Gl. Biol. Neoplasia 3 (3), 259–273. 10.1023/a:1018711410270 10819513

[B62] Mather I. H. Masedunskas A. Chen Y. Weigert R. (2019). Symposium review: intravital imaging of the lactating mammary gland in live mice reveals novel aspects of milk-lipid secretion. J. Dairy Sci. 102 (3), 2760–2782. 10.3168/jds.2018-15459 30471915 PMC7094374

[B63] McManaman J. L. (2012). Milk lipid secretion: recent biomolecular aspects. Biomol. Concepts 3 (6), 581–591. 10.1515/bmc-2012-0025 24605173 PMC3941198

[B64] McManaman J. L. Palmer C. A. Wright R. M. Neville M. C. (2002). Functional regulation of xanthine oxidoreductase expression and localization in the mouse mammary gland: evidence of a role in lipid secretion. J. Physiology 545 (2), 567–579. 10.1113/jphysiol.2002.027185 PMC2290700 12456835

[B65] McNeilly A. S. Robinson I. C. Houston M. J. Howie P. W. (1983). Release of oxytocin and prolactin in response to suckling. Br. Med. J. Clin. Res. ed) 286 (6361), 257–259. 10.1136/bmj.286.6361.257 PMC1546473 6402061

[B66] Monks J. Dzieciatkowska M. Bales E. S. Orlicky D. J. Wright R. M. McManaman J. L. (2016). Xanthine oxidoreductase mediates membrane docking of milk-fat droplets but is not essential for apocrine lipid secretion. J. Physiology 594 (20), 5899–5921. 10.1113/JP272390 PMC5063925 27357166

[B67] Monks J. Ladinsky M. S. McManaman J. L. (2020). Organellar contacts of milk lipid droplets. Contact 3, 251525641989722. 10.1177/2515256419897226 PMC7105144 32232194

[B68] Monks J. Orlicky D. J. Libby A. E. Dzieciatkowska M. Ladinsky M. S. McManaman J. L. (2022). Perilipin-2 promotes lipid droplet-plasma membrane interactions that facilitate apocrine lipid secretion in secretory epithelial cells of the mouse mammary gland. Front. Cell Dev. Biol. 10, 958566. 10.3389/fcell.2022.958566 36158190 PMC9500548

[B69] Neville M. C. Demerath E. W. Hahn-Holbrook J. Hovey R. C. Martin-Carli J. McGuire M. A. (2023). Parental factors that impact the ecology of human mammary development, milk secretion, and milk composition—a report from “Breastmilk Ecology: Genesis of Infant Nutrition (BEGIN)” Working Group 1. Am. J. Clin. Nutr. 117, S11–S27. 10.1016/j.ajcnut.2022.11.026 37173058 PMC10232333

[B70] Neville M. C. Keller R. P. Seacat J. Casey C. E. Allen J. C. Archer P. (1984). Studies on human lactation. I. Within-feed and between-breast variation in selected components of human milk. Am. J. Clin. Nutr. 40 (3), 635–646. 10.1093/ajcn/40.3.635 6475828

[B71] Nommsen-Rivers L. Black M. M. Christian P. Groh-Wargo S. Heinig M. J. Israel-Ballard K. (2023). An equitable, community-engaged translational framework for science in human lactation and infant feeding—a report from “Breastmilk Ecology: Genesis of Infant Nutrition (BEGIN)” Working Group 5. Am. J. Clin. Nutr. 117, S87–S105. 10.1016/j.ajcnut.2023.01.020 37173062 PMC10356563

[B72] Oftedal O. T. (Editor) (1984). Milk composition, milk yield and energy output at peak lactation: a comparative review.

[B73] Oftedal O. T. (2012). The evolution of milk secretion and its ancient origins. Animal 6 (3), 355–368. 10.1017/S1751731111001935 22436214

[B74] Ogg S. L. Weldon A. K. Dobbie L. Smith A. J. H. Mather I. H. (2004a). Expression of butyrophilin (Btn1a1) in lactating mammary gland is essential for the regulated secretion of milk–lipid droplets. Proc. Natl. Acad. Sci. 101 (27), 10084–10089. 10.1073/pnas.0402930101 15226505 PMC454168

[B75] Ogg S. L. Weldon A. K. Dobbie L. Smith A. J. H. Mather I. H. (2004b). Expression of butyrophilin (Btn1a1) in lactating mammary gland is essential for the regulated secretion of milk–lipid droplets. Proc. Natl. Acad. Sci. U. S. A. 101 (27), 10084–10089. 10.1073/pnas.0402930101 15226505 PMC454168

[B76] Ozeki S. Cheng J. Tauchi-Sato K. Hatano N. Taniguchi H. Fujimoto T. (2005). Rab18 localizes to lipid droplets and induces their close apposition to the endoplasmic reticulum-derived membrane. J. Cell Sci. 118 (12), 2601–2611. 10.1242/jcs.02401 15914536

[B77] Patton S. Huston G. E. (1986). A method for isolation of milk fat globules. Lipids 21 (2), 170–174. 10.1007/BF02534441 3959776

[B78] Patton S. Huston G. E. (1988). Incidence and characteristics of cell pieces on human milk fat globules. Biochimica Biophysica Acta (BBA) - General Subj. 965 (2), 146–153. 10.1016/0304-4165(88)90050-5 3365450

[B79] Raiten D. J. Steiber A. L. Papoutsakis C. Rozga M. Handu D. Proaño G. V. (2023). The “breastmilk ecology: Genesis of infant nutrition (BEGIN)” Project – executive summary. Am. J. Clin. Nutr. 117, S1–S10. 10.1016/j.ajcnut.2022.12.020 37173057 PMC10356555

[B80] Reinhardt T. A. Lippolis J. D. (2008). Developmental changes in the milk fat globule membrane proteome during the transition from colostrum to milk. J. Dairy Sci. 91 (6), 2307–2318. 10.3168/jds.2007-0952 18487653

[B81] Robenek H. Hofnagel O. Buers I. Lorkowski S. Schnoor M. Robenek M. J. (2006). Butyrophilin controls milk fat globule secretion. Proc. Natl. Acad. Sci. 103 (27), 10385–10390. 10.1073/pnas.0600795103 16801554 PMC1502467

[B82] Russell T. D. Schaack J. Orlicky D. J. Palmer C. Chang B. H.-J. Chan L. (2011). Adipophilin regulates maturation of cytoplasmic lipid droplets and alveolae in differentiating mammary glands. J. Cell Sci. 124 (19), 3247–3253. 10.1242/jcs.082974 21878492 PMC3178452

[B83] Sherman B. T. Hao M. Qiu J. Jiao X. Baseler M. W. Lane H. C. (2022). DAVID: a web server for functional enrichment analysis and functional annotation of gene lists (2021 update). Nucleic Acids Res. 50 (W1), W216–W221. 10.1093/nar/gkac194 35325185 PMC9252805

[B84] Smilowitz J. T. Allen L. H. Dallas D. C. McManaman J. Raiten D. J. Rozga M. (2023). Ecologies, synergies, and biological systems shaping human milk composition—a report from “Breastmilk Ecology: Genesis of Infant Nutrition (BEGIN)” Working Group 2. Am. J. Clin. Nutr. 117, S28–S42. 10.1016/j.ajcnut.2022.11.027 37173059 PMC10356566

[B85] Smith S. J. Cases S. Jensen D. R. Chen H. C. Sande E. Tow B. (2000). Obesity resistance and multiple mechanisms of triglyceride synthesis in mice lacking Dgat. Nat. Genet. 25 (1), 87–90. 10.1038/75651 10802663

[B86] Spertino S. Cipriani V. De Angelis C. Giuffrida M. G. Marsano F. Cavaletto M. (2012). Proteome profile and biological activity of caprine, bovine and human milk fat globules. Mol. Biosyst. 8 (4), 967–974. 10.1039/c2mb05400k 22193558

[B87] Stein O. Stein Y. (1967). Lipid synthesis, intracellular transport, and secretion: II. Electron microscopic radioautographic study of the mouse lactating mammary gland. J. Cell Biol. 34 (1), 251–263. 10.1083/jcb.34.1.251 6033535 PMC2107216

[B88] Stemberger B. H. Patton S. (1981). Relationships of size, intracellular location, and time required for secretion of milk fat droplets. J. Dairy Sci. 64 (3), 422–426. 10.3168/jds.s0022-0302(81)82588-x

[B89] Stemberger B. H. Walsh R. M. Patton S. (1984). Morphometric evaluation of lipid droplet associations with secretory vesicles, mitochondria and other components in the lactating cell. Cell Tissue Res. 236 (2), 471–475. 10.1007/BF00214252 6733773

[B90] Suburu J. Shi L. Wu J. Wang S. Samuel M. Thomas M. J. (2014). Fatty acid synthase is required for mammary gland development and milk production during lactation. Am. J. Physiology-Endocrinology Metabolism 306 (10), E1132–E1143. 10.1152/ajpendo.00514.2013 PMC4116404 24668799

[B91] Thum C. Wall C. Day L. Szeto I. M. Y. Li F. Yan Y. (2022). Changes in human milk fat globule composition throughout lactation: a review. Front. Nutr. 9, 835856. 10.3389/fnut.2022.835856 35634409 PMC9137899

[B92] Victora C. G. Bahl R. Barros A. J. D. França G. V. A. Horton S. Krasevec J. (2016). Breastfeeding in the 21st century: epidemiology, mechanisms, and lifelong effect. Lancet 387 (10017), 475–490. 10.1016/S0140-6736(15)01024-7 26869575

[B93] Vorbach C. Scriven A. Capecchi M. R. (2002). The housekeeping gene xanthine oxidoreductase is necessary for milk fat droplet enveloping and secretion: gene sharing in the lactating mammary gland. Genes and Dev. 16 (24), 3223–3235. 10.1101/gad.1032702 12502743 PMC187506

[B94] Walther T. C. Jr. Farese R. V. Jr (2012). Lipid droplets and cellular lipid metabolism. Annu. Rev. Biochem. 81 (1), 687–714. 10.1146/annurev-biochem-061009-102430 22524315 PMC3767414

[B95] Wang W. Lv N. Zhang S. Shui G. Qian H. Zhang J. (2012). Cidea is an essential transcriptional coactivator regulating mammary gland secretion of milk lipids. Nat. Med. 18 (2), 235–243. 10.1038/nm.2614 22245780

[B96] Wooding F. B. Kemp P. (1975). Ultrastructure of the milk fat globule membrane with and without triglyceride. Cell Tissue Res. 165 (1), 113–127. 10.1007/BF00222804 1203969

[B20] Wooding FBP (1971). The mechanism of secretion of the milk fat globule. J. Cell Sci. 9 (3), 805–821. 10.1242/jcs.9.3.805 5148018

[B21] Wooding FBP (1973). Formation of the milk fat globule membrane without participation of the plasmalemma. J. Cell Sci. 13 (1), 221–235. 10.1242/jcs.13.1.221 4729936

[B22] Wooding FBP (2023). Mammary lipid secretion: a reassessment. J. Dairy Res. 90 (1), 28–37. 10.1017/s0022029923000109 36911923

[B97] Wu C. C. Howell K. E. Neville M. C. Yates J. R. III McManaman J. L. (2000). Proteomics reveal a link between the endoplasmic reticulum and lipid secretory mechanisms in mammary epithelial cells. ELECTROPHORESIS 21 (16), 3470–3482. 10.1002/1522-2683(20001001)21:16<3470::AID-ELPS3470>3.0.CO;2-G 11079566

[B98] Wu L. Zhou L. Chen C. Gong J. Xu L. Ye J. (2014). Cidea controls lipid droplet fusion and lipid storage in brown and white adipose tissue. Sci. China Life Sci. 57 (1), 107–116. 10.1007/s11427-013-4585-y 24369348

[B99] Xu D. Li Y. Wu L. Li Y. Zhao D. Yu J. (2018). Rab18 promotes lipid droplet (LD) growth by tethering the ER to LDs through SNARE and NRZ interactions. J. Cell Biol. 217 (3), 975–995. 10.1083/jcb.201704184 29367353 PMC5839781

[B100] Yang M. Cong M. Peng X. Wu J. Wu R. Liu B. (2016). Quantitative proteomic analysis of milk fat globule membrane (MFGM) proteins in human and bovine colostrum and mature milk samples through iTRAQ labeling. Food and Funct. 7 (5), 2438–2450. 10.1039/c6fo00083e 27159491

[B101] Yang Y. Zheng N. Zhao X. Zhang Y. Han R. Ma L. (2015). Proteomic characterization and comparison of mammalian milk fat globule proteomes by iTRAQ analysis. J. Proteomics 116, 34–43. 10.1016/j.jprot.2014.12.017 25576853

[B102] Zappa F. Venditti R. De Matteis M. A. (2017). TRAPPing Rab18 in lipid droplets. EMBO J. 36 (4), 394–396. 10.15252/embj.201696287 28130247 PMC5694943

[B103] Zhang X. Jiang B. Ji C. Li H. Yang L. Jiang G. (2021). Quantitative label-free proteomic analysis of milk fat globule membrane in donkey and human milk. Front. Nutr. 8, 670099. 10.3389/fnut.2021.670099 34239890 PMC8258387

[B104] Zhao H. Ahirwar D. K. Oghumu S. Wilkie T. Powell Catherine A. Nasser M. W. (2016). Endothelial Robo4 suppresses breast cancer growth and metastasis through regulation of tumor angiogenesis. Mol. Oncol. 10 (2), 272–281. 10.1016/j.molonc.2015.10.007 26778715 PMC4739522

[B105] Zhao L. Ke H. Xu H. Wang G.-D. Zhang H. Zou L. (2020). TDP-43 facilitates milk lipid secretion by post-transcriptional regulation of Btn1a1 and Xdh. Nat. Commun. 11 (1), 341. 10.1038/s41467-019-14183-1 31953403 PMC6969145

[B106] Zhou Y. Zhou B. Pache L. Chang M. Khodabakhshi A. H. Tanaseichuk O. (2019). Metascape provides a biologist-oriented resource for the analysis of systems-level datasets. Nat. Commun. 10 (1), 1523. 10.1038/s41467-019-09234-6 30944313 PMC6447622

