# Supplementary material for: Comparative proteomic analysis of human milk fat globules and paired membranes and mouse milk fat globules identifies core cellular systems contributing to mammary lipid trafficking and secretion
Source: Front Mol Biosci. 2023 Dec 18;10:1259047. doi: 10.3389/fmolb.2023.1259047 (PMC10759240; doi:10.3389/fmolb.2023.1259047)
Supplement: Supplementary file 1 [file Presentation1.pptx]

## Slide 1
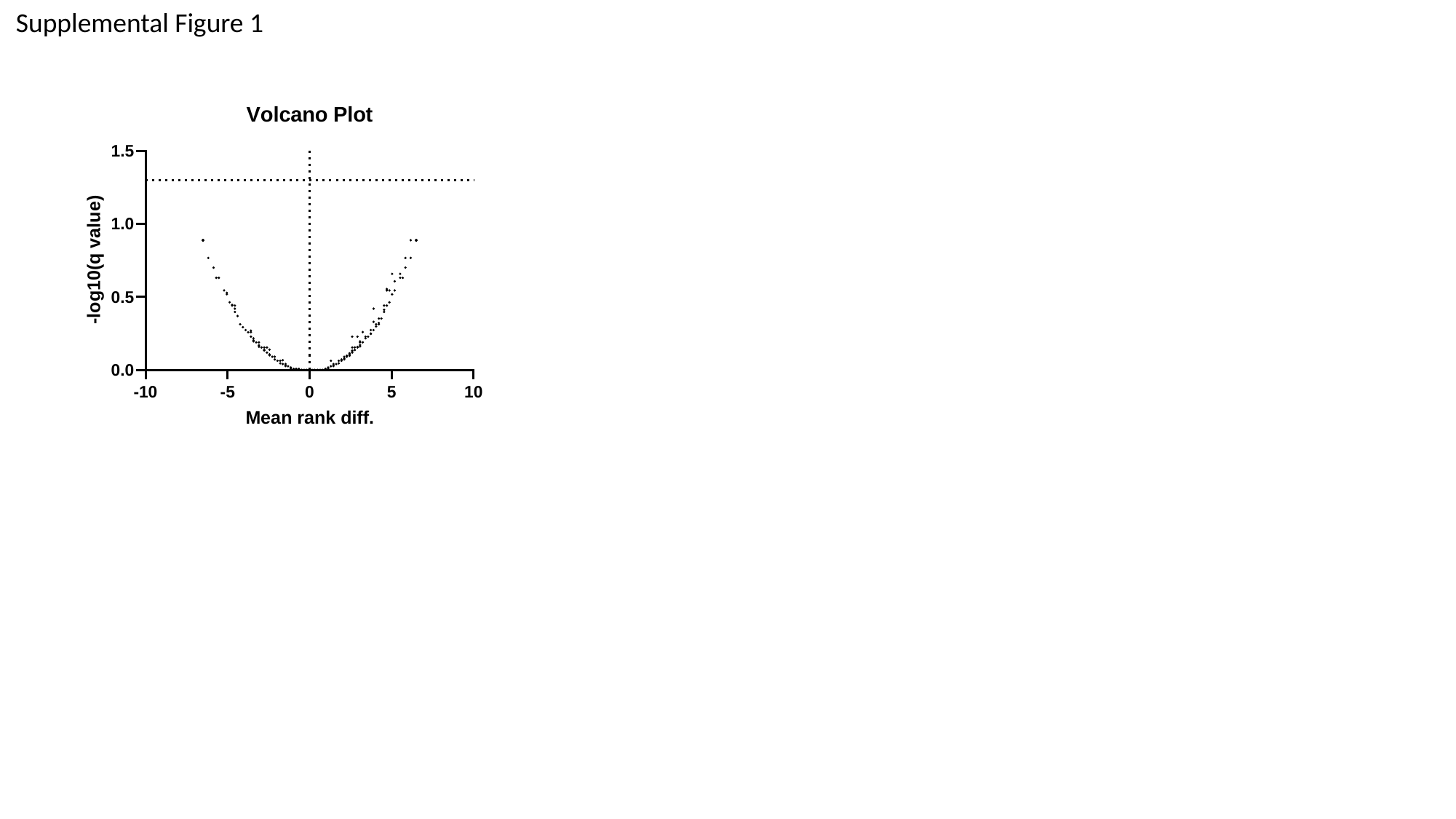

Supplemental Figure 1

## Slide 2
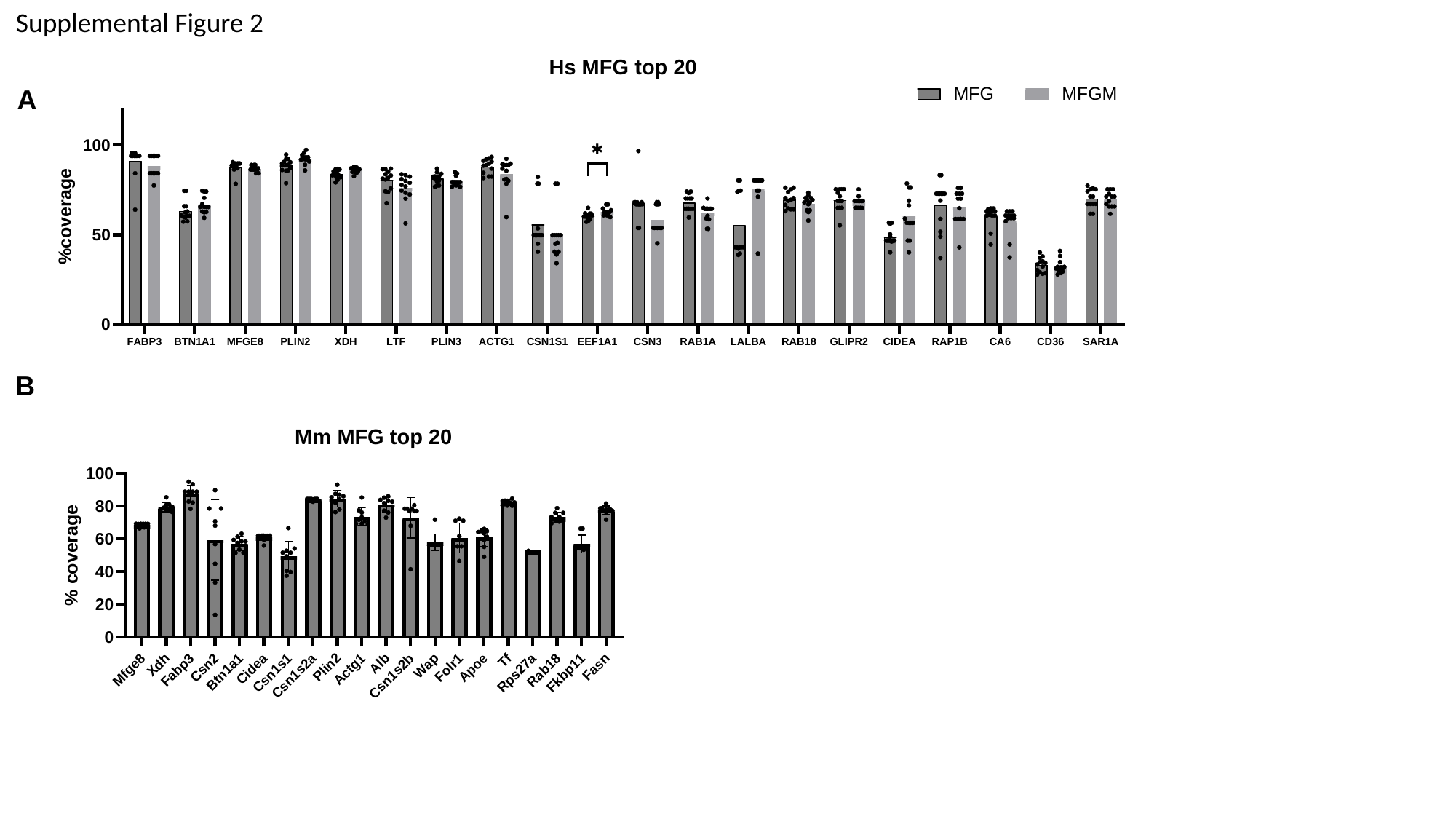

Supplemental Figure 2
A
B

## Slide 3
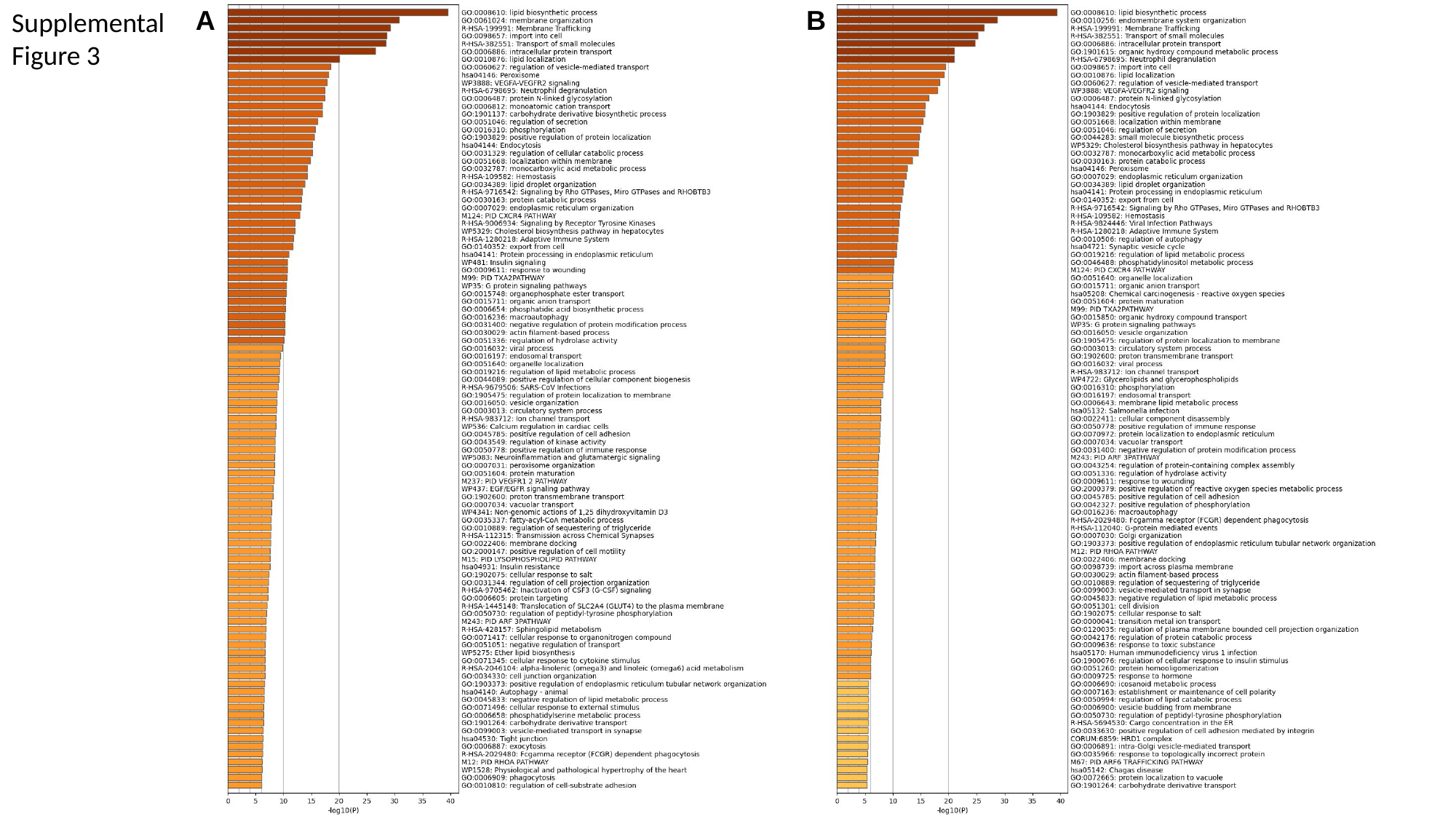

Supplemental
Figure 3
A
B

## Slide 4
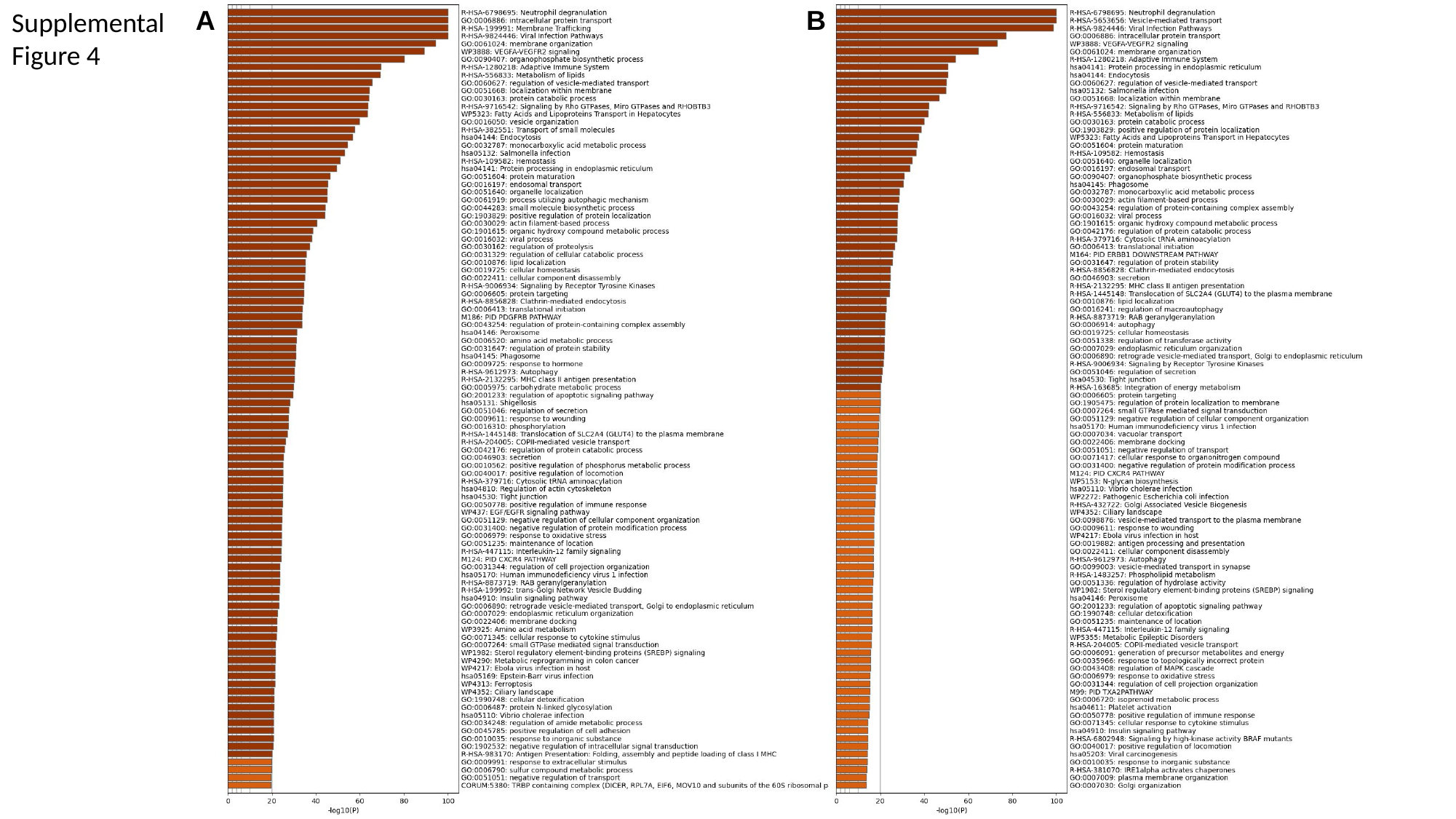

Supplemental
Figure 4
A
B

## Slide 5
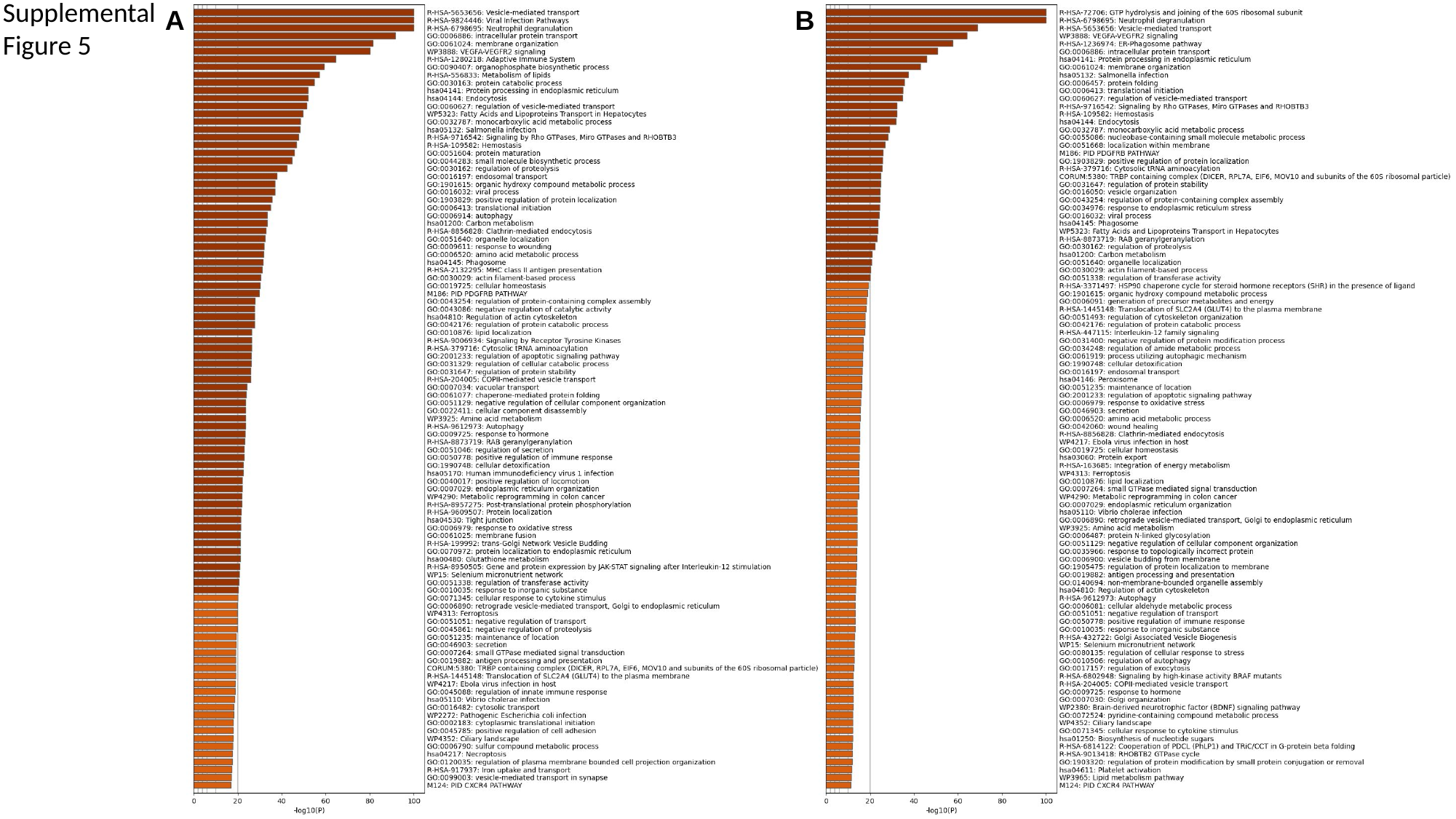

Supplemental
Figure 5
A
B

## Slide 6
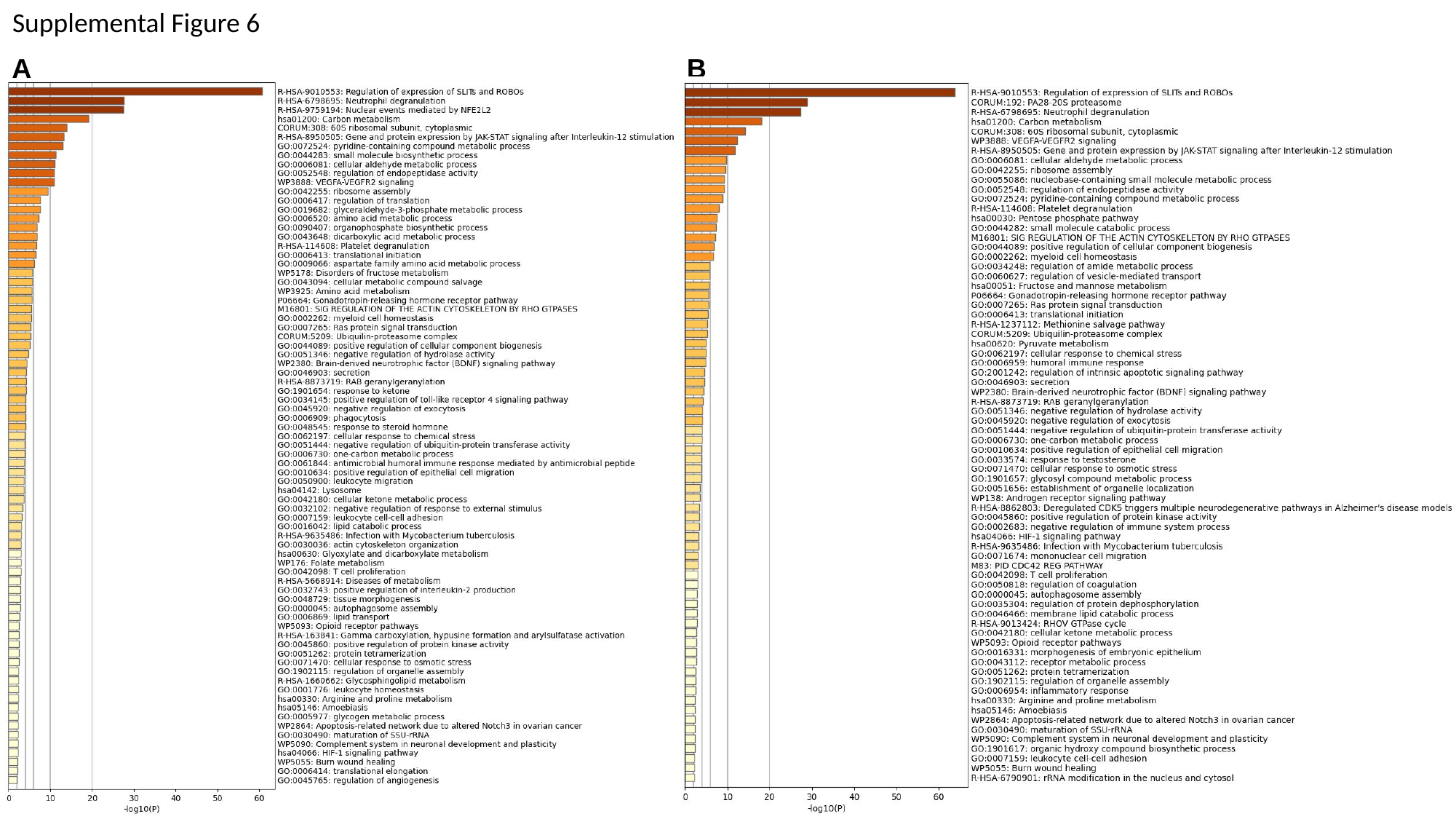

Supplemental Figure 6
A
B

## Slide 7
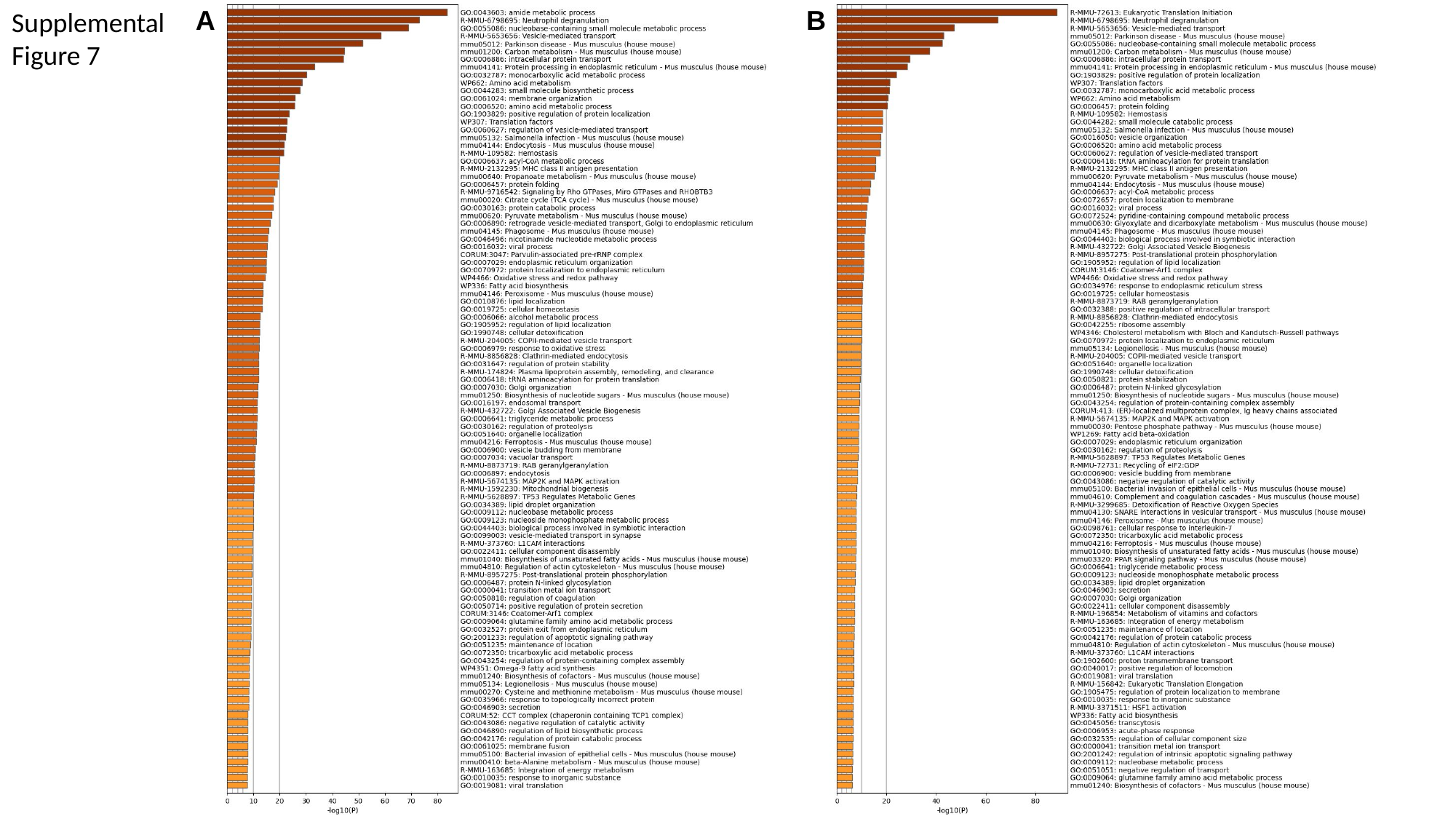

Supplemental
Figure 7
A
B

## Slide 8
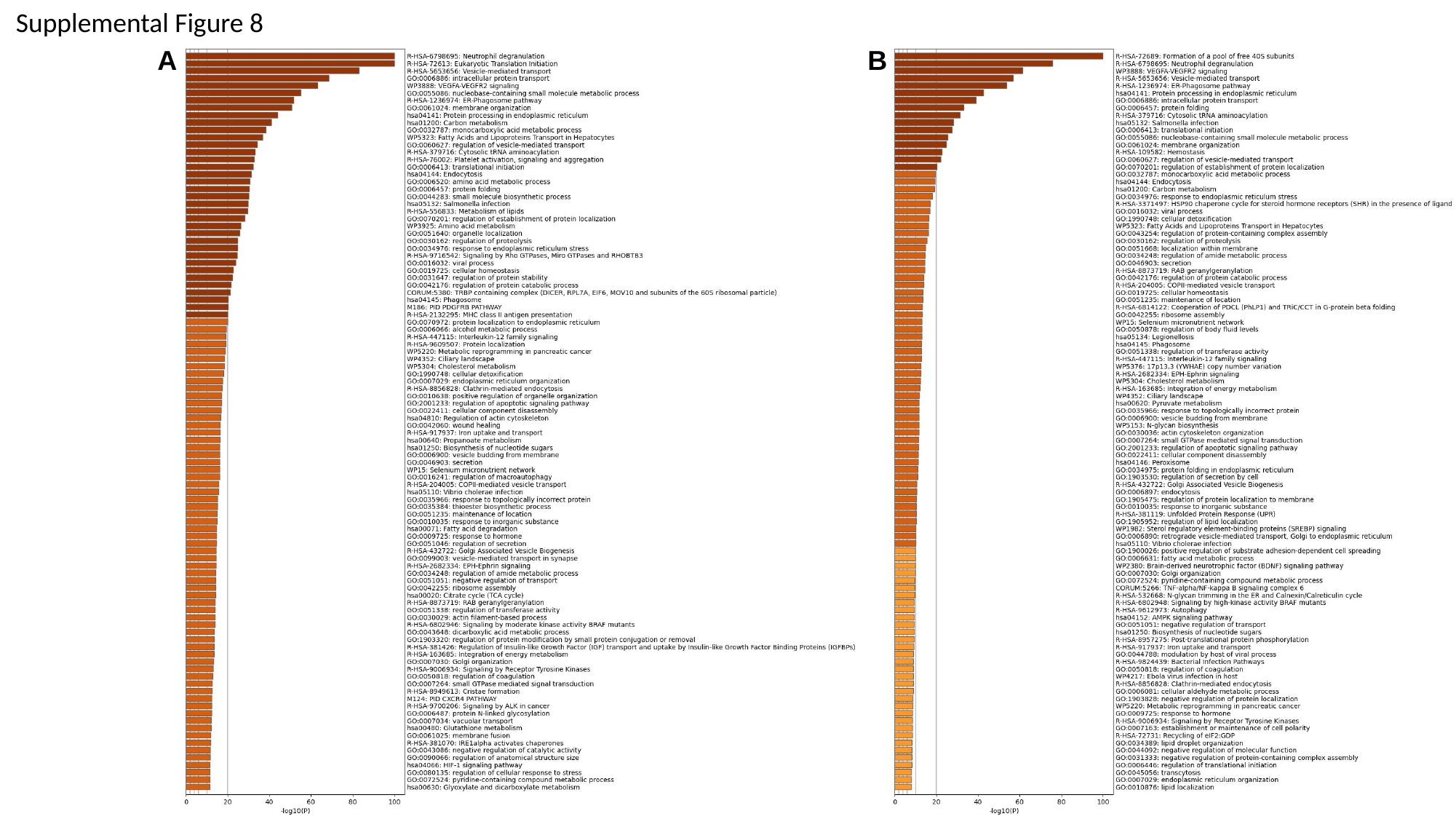

Supplemental Figure 8
A
B

## Slide 9
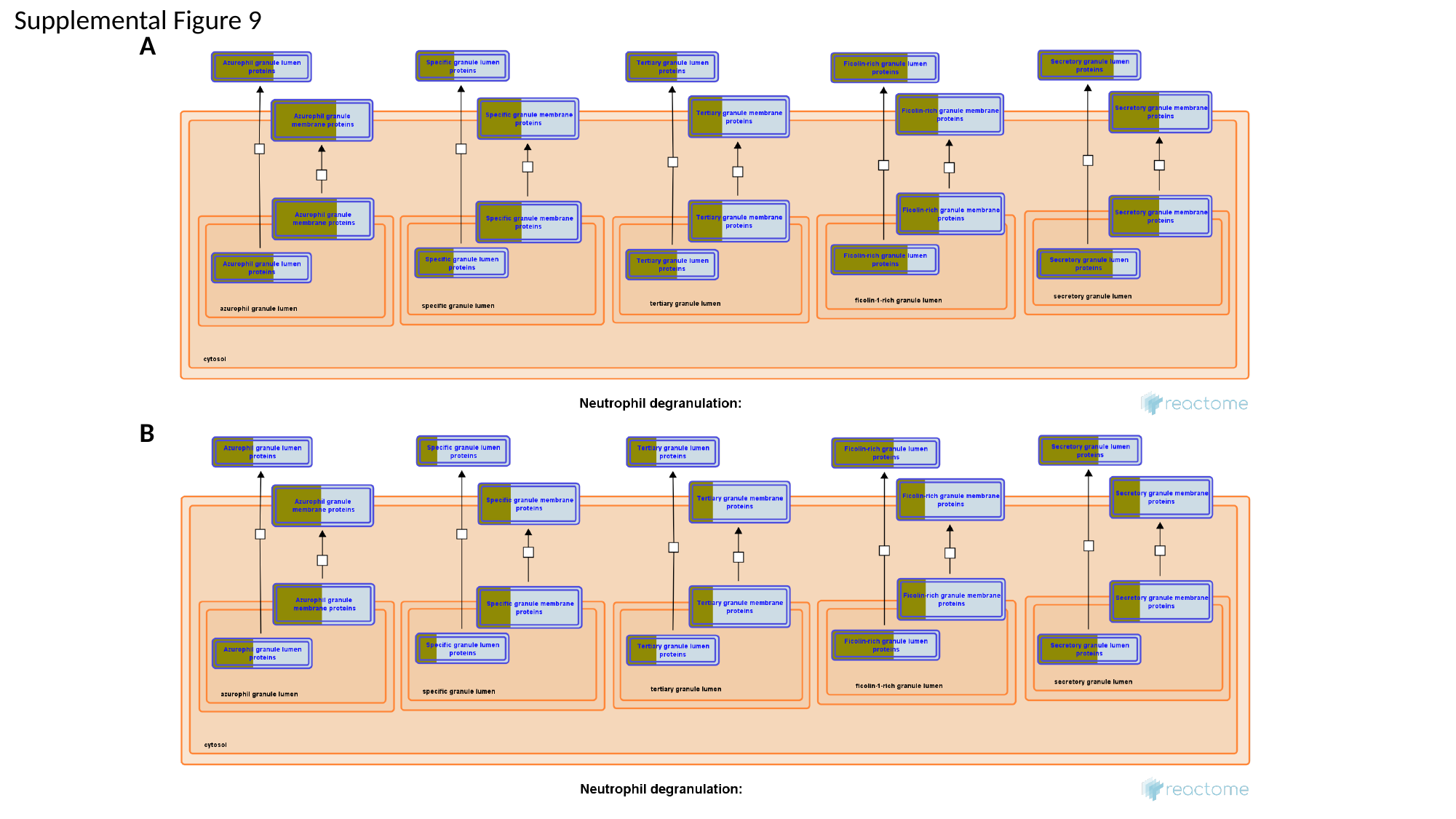

Supplemental Figure 9
A
B

## Slide 10
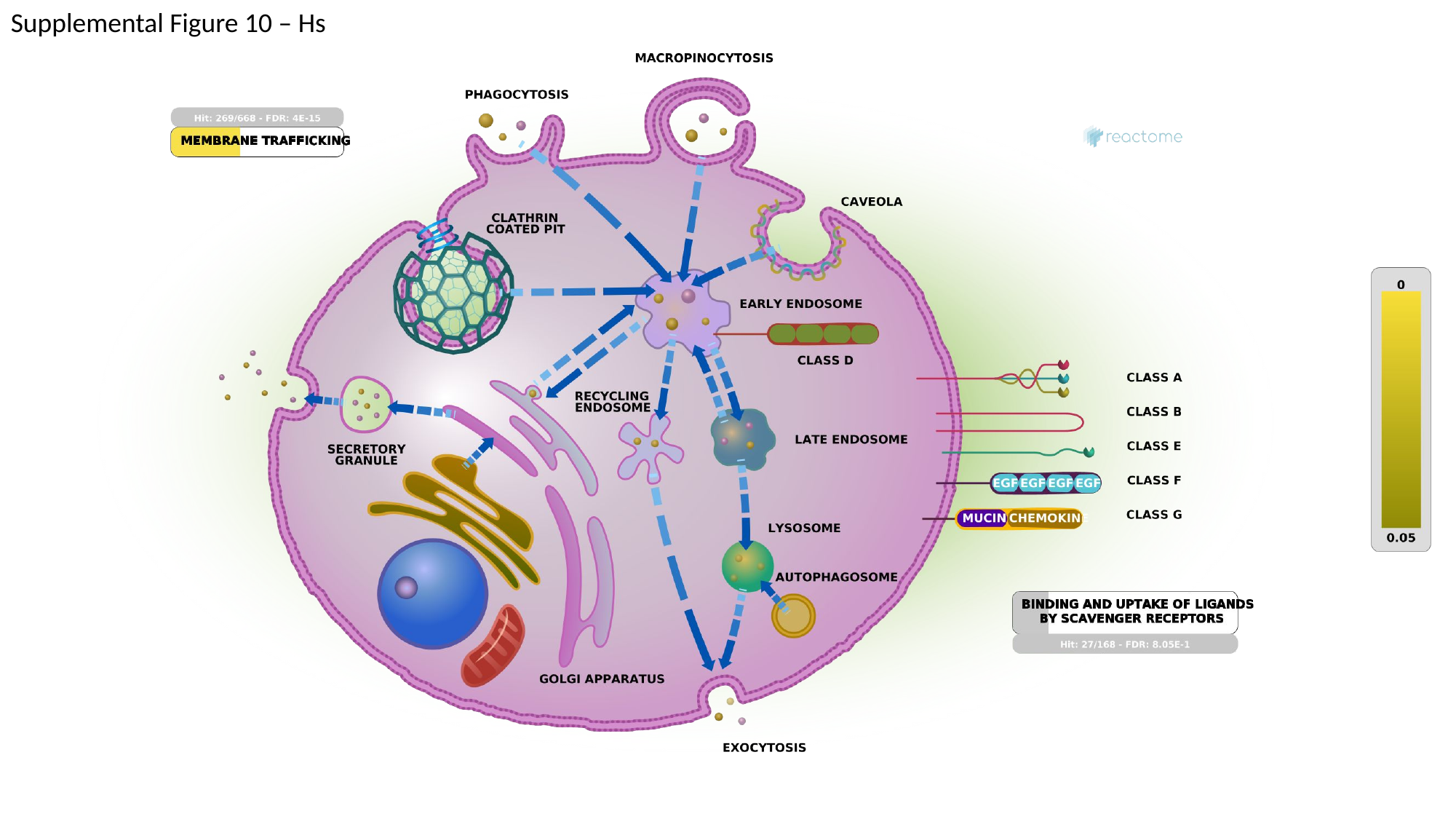

Supplemental Figure 10 – Hs

## Slide 11
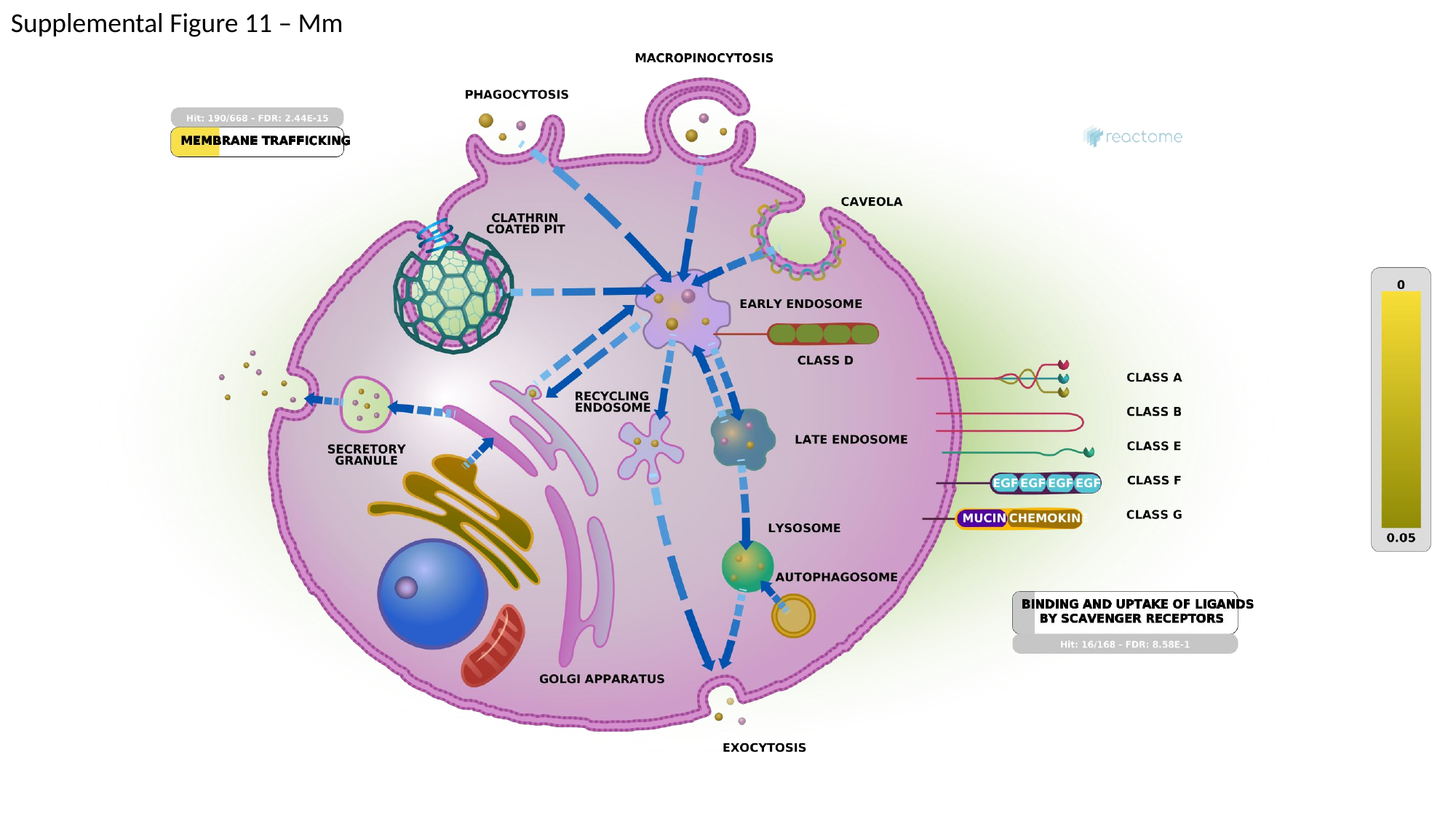

Supplemental Figure 11 – Mm

## Slide 12
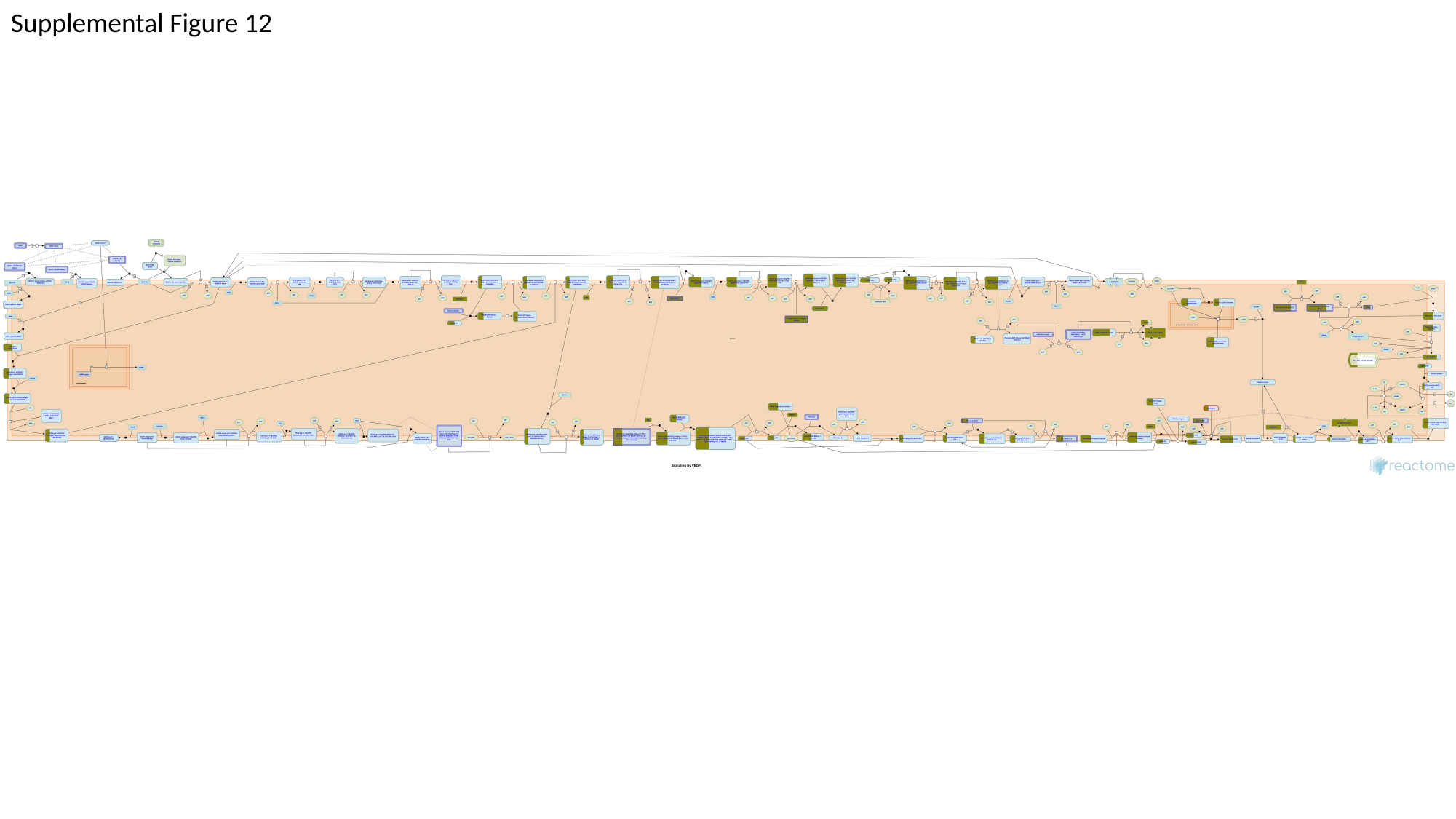

Supplemental Figure 12
